# Protein interaction disruption in cancer

**DOI:** 10.1186/s12885-019-5532-5

**Published:** 2019-04-23

**Authors:** Matthew Ruffalo, Ziv Bar-Joseph

**Affiliations:** 1 Computational Biology Department, School of Computer Science, Carnegie Mellon University, 5000 Forbes Avenue, Pittsburgh, PA, 15213 USA; 2 Machine Learning Department, School of Computer Science, Carnegie Mellon University, 5000 Forbes Avenue, Pittsburgh, PA, 15213 USA

**Keywords:** TCGA, Breast cancer, Feature construction, Protein interaction

## Abstract

**Background:**

Most methods that integrate network and mutation data to study cancer focus on the effects of genes/proteins, quantifying the effect of mutations or differential expression of a gene and its neighbors, or identifying groups of genes that are significantly up- or down-regulated. However, several mutations are known to disrupt specific protein-protein interactions, and network dynamics are often ignored by such methods. Here we introduce a method that allows for predicting the disruption of specific interactions in cancer patients using somatic mutation data and protein interaction networks.

**Methods:**

We extend standard network smoothing techniques to assign scores to the edges in a protein interaction network in addition to nodes. We use somatic mutations as input to our modified network smoothing method, producing scores that quantify the proximity of each edge to somatic mutations in individual samples.

**Results:**

Using breast cancer mutation data, we show that predicted edges are significantly associated with patient survival and known ligand binding site mutations. In-silico analysis of protein binding further supports the ability of the method to infer novel disrupted interactions and provides a mechanistic explanation for the impact of mutations on key pathways.

**Conclusions:**

Our results show the utility of our method both in identifying disruptions of protein interactions from known ligand binding site mutations, and in selecting novel clinically significant interactions.Supporting website with software and data: https://www.cs.cmu.edu/~mruffalo/mut-edge-disrupt/.

**Electronic supplementary material:**

The online version of this article (10.1186/s12885-019-5532-5) contains supplementary material, which is available to authorized users.

## Background

The impact of DNA mutations on the severity and progress of cancer has been a long standing focus for systems biology. On the one hand, several mutations to key genes were shown to play a critical role in cancer development and progression [1–7]. However, most mutations observed in cancer patients are unique, seen only in the individual in which they were observed, making it hard to determine their impact and to differentiate between causal and driver mutations [8, 9]. To address this issue, several network analysis methods have been used to aggregate the impact of mutations within and across patients [10, 11]. These methods operate under the assumptions that genes in a specific neighborhood of an interaction graph likely share a function or a pathway and so mutations in these genes, even if unique, may inform us about the importance of that pathway to the specific type of cancer being studied. An example of such network based methods is network smoothing, which fuses network structure with prior knowledge, and produces a measure for each node that respects both the input data and the structure of the network [12]. Such smoothing methods are widely used, with applications ranging from identification of cancer genes [13, 14], identification of gained/lost cellular functions [15] and more [12].

Network smoothing methods are commonly used to quantify the proximity of each node in the network to a set of nodes of interest, e.g. genes that are mutated or differentially expressed in a sample. While successful in identifying cancer genes and pathways, these methods are limited to using a static network that is shared between samples, and are not designed to handle dynamic effects (such as changes in interactions between samples). Mutations may disrupt interactions between proteins through a variety of mechanisms: alteration of protein structure impacting its function [16–18], affecting the ability of a protein to bind DNA [19–22], impacting the regulation of a gene, affecting its translation or degradation efficiency [23–25] and more. Most work utilizing protein-protein interaction networks in cancer do not adjust the networks based on such individual mutation information [26–28]. Thus, there is a need for methods that can perform comprehensive genome-wide prediction of protein interaction disruption and can determine the impact of such disruption on the resulting pathways and networks.

To enable the identification of mutations that significantly alter edges in the network we extended network smoothing algorithms to smooth not just node values but also edge (interaction) values. We do this by adding a set of nodes that represent the edges, assigning an initial value to each of these nodes and then performing network smoothing on the (much larger) network. This network adjustment has some conceptual similarities with other graph operations such as graph powers, in which transitive edges are added to an existing network; double graphs, in which a graph is duplicated and “cross” edges are added for each original edge; and line graphs, which represent edges of the original graph as nodes. We discuss the algorithmic and run time implications of the combined node and edge smoothing method. We next applied our method to study over a thousand mutation profiles from TCGA breast cancer patients. As we show, the network smoothing method was able to prioritize a subset of the edges, based on the mutation information alone, that were both better at predicting survival across patients and correctly associated with known ligand binding mutations. We discuss some of the top interactions identified by the method and show that these indeed include mainly known cancer related genes. Finally, for the subset of the predicted edges for which we could find structural information we tested the impact of the mutation on the specific interaction predicted and show that the *R*^2^ correlation between the predicted and actual impact is high.

## Methods

### Pre-processing the omics data

We obtained somatic mutation and clinical data from breast cancer (BRCA) samples in TCGA [29], which we used to construct features for prediction of interaction disruption.

We constructed a binary mutation matrix *M*, with samples as rows and genes as columns. We use *C*(*A*) to denote the set of column labels of matrix *A*, so that e.g. *C*(*M*) is the set of genes that appear in the TCGA somatic mutation data. Similarly, we define *R*(*A*) as the set of row labels of matrix *A*, corresponding to the distinct samples (individuals) present in each data set.

The mutation matrices *M* are defined as

1 $$ M[i, j] = \left\{ \begin{array}{ll} 1 & \text{if gene \textit{j} is mutated in sample \textit{i}}, \\ 0 & \text{otherwise} \end{array}\right.  $$

The TCGA BRCA data includes somatic mutations in 22,232 genes across 1081 samples, including missense mutations, nonsense mutations, frame shifts, and in-frame deletions and insertions. In addition to the condition specific omics data we also use general interaction datasets. Our primary results use the HIPPIE protein-protein interaction network [30] (version 2.0, released 2016-06-24), which contains confidence scores for 318,757 interactions between 17,204 proteins. We also evaluate our method using the STRING network (v10.5), using all edges included in the downloadable version of that network: 4,724,503 edges between 17,179 nodes. Edges in the STRING network must have a weight of at least 0.15 to be included in the downloadable version of the network; we use all available edges in this version of STRING. Note that the network smoothing procedure allows using these edges in a way that respects the degree of confidence in those protein interaction – low-weight edges contribute less to the result of the network smoothing operation (Additional file 1: Supporting Methods). Results using the STRING network are shown in Additional file 1.

### Network construction and initial edge scores

Given an original PPI network *G*=(*V, E*,*w*), with *V* as the set of proteins, *E* as the set of edges, and edge weights *w*(*u, v*) on every edge {*u, v*}∈*E*, we create an adjusted network *G*^′^=(*V*^′^,*E*^′^,*w*^′^). With *Adj*_*G*_[*v*] as the adjacency list of *v* in the network *G*, we define *V*^′^ and *E*^′^:

2 $$ \begin{aligned} V' =& V \cup \left\{uv : \{u, v\} \in E\right\} \\ E' =& \left\{\{u, uv\} : u \in V \wedge v \in {Adj}_{G}[v]\right\} \end{aligned}  $$

**Fig. 1:**
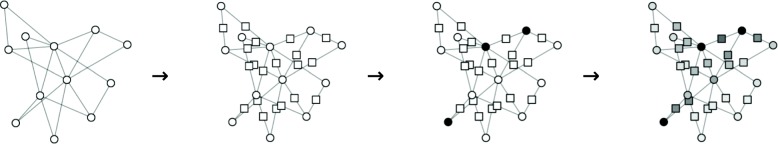
Simulation of the edge smoothing procedure. From left to right: the original protein-protein interaction network, the adjusted network with dummy nodes (squares) for each protein-protein edge, somatic mutations shown as black nodes, and the result of the network smoothing procedure applied to the adjusted network with dummy nodes. White and black nodes in the third panel show assignment of values 0 and 1 (respectively) to nodes, and the fourth panel shows continuous node values in [0,1], denoting the smoothed score for each protein and protein-protein interaction

That is, we add a dummy node *uv* in the middle of each edge {*u, v*}, as shown in Fig. 1. These dummy nodes in *G*^′^ represent edges in *G*, and allow assigning scores to each edge by extending current network smoothing procedures.

We define initial weights for our new edges in *G*^′^ as:

3 $$ w'(u, uv) = w'(uv, v) = \sqrt{w(u, v)}   $$

Protein interaction networks often use edge weights *w*(*u, v*)∈[0,1] to denote the confidence in some edge (*u, v*), and one can naturally define the *reliability* of a path *p*_*st*_ between nodes *s* and *t* as the product of edge weights along this path [31].

4 $$ r(p_{st}) = \prod_{(u, v) \in p_{st}} w(u, v)  $$

Our choice of edge weights $w'(u, uv) = w'(uv, v) = \sqrt {w(u, v)}$ preserves the reliability of any path between two nodes *s* and *t* representing proteins in the network *G*, giving the same reliability $\phantom {\dot {i}\!}r(p_{s^{\prime }t^{\prime }})$ in *G*^′^ (Additional file 1: Supporting Methods). We also evaluate our method using an alternative assignment of edge weights, with *w*^′^(*u, uv*)=*w*^′^(*uv, v*)=*w*(*u, v*)/2 (Additional file 1: Supporting Results).

Once we assign an initial score to edges, we use our adjusted network *G*^′^ to perform a standard network smoothing procedure, as described in the following section.

### Gene set network smoothing

Here we extend the network propagation/smoothing method described in Vanunu et al. [32] that was initially only focused on nodes to smooth edge scores as well. Given a network *G*=(*V, E*,*w*) with *V* as the set of proteins and new nodes for original edges, *E* as the set of edges linking proteins with new edge nodes, edge weights defined in Eq. 3, and a prior knowledge vector *Y*:*V*→[0,1] constructed from somatic mutation status, we compute a function *F*(*v*) that is both smooth over the network and accounts for the prior knowledge about each node. Note that we do not perform this network smoothing procedure directly on the protein-protein interaction network; we compute smoothed node scores for our modified network that contains dummy nodes corresponding to edges in the original network and thus allows for scoring edges as well as nodes (Additional file 1: Supporting Methods).

### Ligand binding site mutations

The mutLBSgeneDB database [33] contains annotations for genes with ligand binding site (LBS) mutations, and we combine these annotations with TCGA somatic mutation data. Of the 1081 TCGA samples with somatic mutation data, 389 have at least one somatic mutation which is contained in the LBS database, and 102 of these samples contain more than one LBS mutation, giving a total of 550 LBS mutations across all samples, in 340 distinct genes. We use these selected ligand binding mutations to evaluate our ranking of interaction edges, in “Ligand binding site edge scoring” section.

### Protein structure alteration prediction

We use protein structures deposited in the RCSB (Research Collaboratory for Structural Bioinformatics) PDB database [34], and perform automated queries to PDB for all ligand binding site mutations in our dataset. We select edges which have a ligand binding site mutation in at least one interacting protein, and for which both interacting proteins have structures in PDB. This produces 143 selected edges, across 24 distinct patients and 98 distinct proteins. For these edges, it is possible, in principle, to use structural alteration prediction to predict binding disruption – though the results of our PDB queries require manual filtering to be usable for this task.

The mutLBSgeneDB database [33] includes specific amino acid substitutions for ligand binding site mutations in TCGA samples. We use the PyMOL tool [35] (version 2.0.7) mutagenesis functionality to simulate the effect of these amino acid substitutions on the relevant protein structures. We then upload structures for these interacting pairs to the ClusPro 2.0 [36] web service to predict protein docking, running two docking prediction jobs for each interacting pair: wild type of both proteins, and the PyMOL-simulated mutated protein structure with wild type of its interacting partner.

## Results

We evaluate our edge scoring method in multiple ways. First, we examine whether high-scoring edges (those that we predict to be more disrupted based on mutational scores) are more predictive of patient survival than random sets of other edges. We then test whether our edge scores show significant agreement with known ligand binding site mutations. Finally we perform simulations of protein docking with and without ligand binding site mutations, and compare our edge scores to a measure of the disruption of specific protein interactions.

### Identification of top scoring edges

To identify mutations impacting network edges we extended network smoothing so that it can produce smoothed scores for *edges* as well. We applied our method to somatic mutation data from TCGA breast invasive carcinoma (BRCA) samples [29]. The dataset contains mutation and survival information for 1081 patients. We use version 2.0 of the HIPPIE protein interaction network [30] to construct an expanded interaction network. The HIPPIE 2.0 network *H*=(*V*_*H*_,*E*_*H*_) has |*E*_*H*_|=314727 edges between |*V*_*H*_|=17204 nodes (genes), and our adjusted network *H*^′^=(*VH*′,*EH*′) has |*VH*′|=|*V*_*H*_|+|*E*_*H*_|=331931 nodes connected by |*EH*′|=2|*E*|=629454 edges. The STRING v10.5 network *S*=(*V*_*S*_,*E*_*S*_) likewise contains |*E*_*S*_|=4724503 edges between |*V*_*S*_|=17179 nodes, and our adjusted network *S*^′^=(*VS*′,*ES*′) contains |*VS*′|=4741682 nodes and |*ES*′|=9449006 edges.

**Fig. 2:**
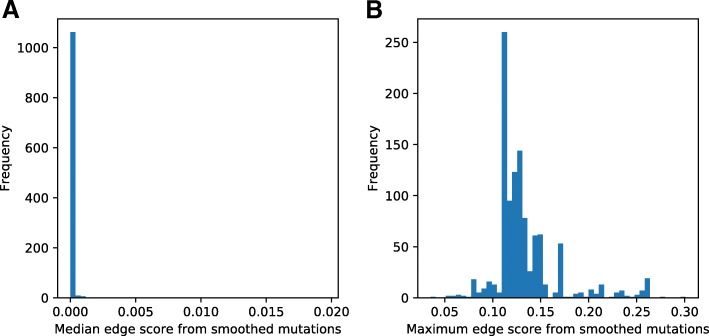
Histograms of propagated edge scores. For each patient, scores are collapsed across all edges by computing the median or maximum edge score in that patient. **a** shows the distribution of the median edge score in each patient, and **b** shows the distribution of the maximum edge score in each patient

For each sample in the TCGA BRCA data, we compute a smoothed mutational score for all nodes in *H*^′^ or *S*^′^, using somatic mutations to assign initial labels to nodes. This produces a continuous score *m*[*v*]∈[0,1] for each *v*∈*VH*′ or $V^{\prime }_{S}$, which represents the proximity of that protein or interaction to somatic mutations in that patient. For each patient, we compute the median and maximum score across all edges, and plot histograms of the median and maximum for the HIPPIE network (Fig. 2) and STRING network (Additional file 1: Figure S12).

### Evaluation of edge scoring procedure

To evaluate the scores assigned to edges, and to determine if they indeed highlight key mutations that impact disease progression, we used several complementary information sources. We first examined the association between our propagated edge scores and patient survival. For this, we fit a univariate Cox regression model for each edge in the network, relating patient survival to each edge’s propagated mutation scores across patients. Cox models are commonly used in survival analysis, as these allow for dealing with censored survival data, in which exact survival times are known for some samples, but only lower bounds are known for others (e.g. if the patient was alive at their last follow-up, but no further information is known) [37, 38]. We compute the *R*^2^ goodness-of-fit value for the Cox model fit to each edge, and evaluate the difference in survival fits between high-scoring edges and random selections of the remaining edges.

**Fig. 3:**
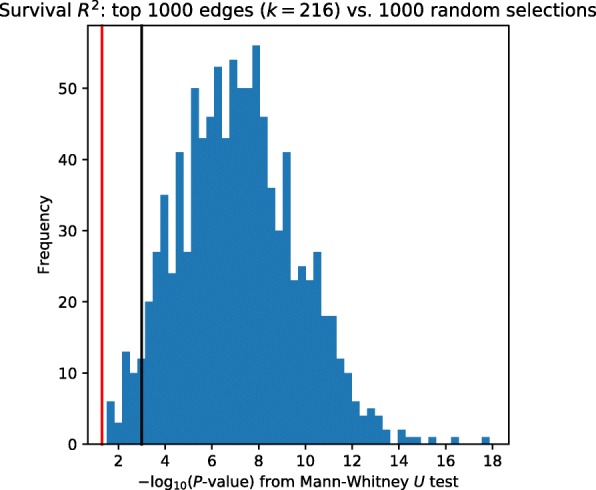
Histogram of Mann-Whitney *U* test *P*-values, comparing survival *R*^2^ values for top-scoring edges and 1000 sets of randomly-selected edges. The red vertical line shows *P*=0.05, the black vertical line shows *P*=0.001. *P*-values from the 1000 M–W tests are transformed to − log10-scale

We collapse propagated edge values across patients by considering the 80^th^ decile of propagated mutation scores for that edge, i.e. the ⌊1081/5⌋=216^th^-highest score for that edge across any patient. These 80^th^-decile scores produce a measure of network proximity of each edge to somatic mutations in at least 20% of patients, and we use these scores to produce a global ranking of edges across all patients. We test whether the top 1000 edges have significantly higher *R*^2^ values than a random sample of 1000 edges. For each of the random sets we perform a Mann-Whitney *U* test to determine whether our top edges have higher *R*^2^ values than randomly chosen edges (Fig. 3). As can be seen, when compared to most random selections top scoring edges obtain a significantly higher *R*^2^ value with survival indicating that mutations related to these edges indeed impact disease progression. We repeated this analysis with alternative edge scores *w*^′^=*w*/2 and using the STRING network (Additional file 1: S10 and S16). In both additional of this survival analysis, we again see that high-scoring edges show a significantly higher *R*^2^ value when compared to random selections.

### Ligand binding site edge scoring

While survival analysis provides some evidence for the relevance of the high scoring edges, it does not provide any mechanistic explanation or support for these scores. To determine the relevance of the high scoring edge mutations to the interactions of the edge proteins (the two proteins on either side of the edge) we looked at a database of ligand binding site (LBS) mutations [33]. This database contains annotations for known ligand binding site mutations across the human genome, including additional cross-database references such as GO process terms, conservation information, and more. Every (gene,amino acid substitution) pair in this database is known to affect a ligand binding site in the protein product of that gene; we extract these pairs and use them to identify all somatic mutations in the TCGA BRCA cohort that are also listed in the mutLBSgeneDB database, allowing us to identify edges which are incident to these ligand binding site mutations.

**Fig. 4:**
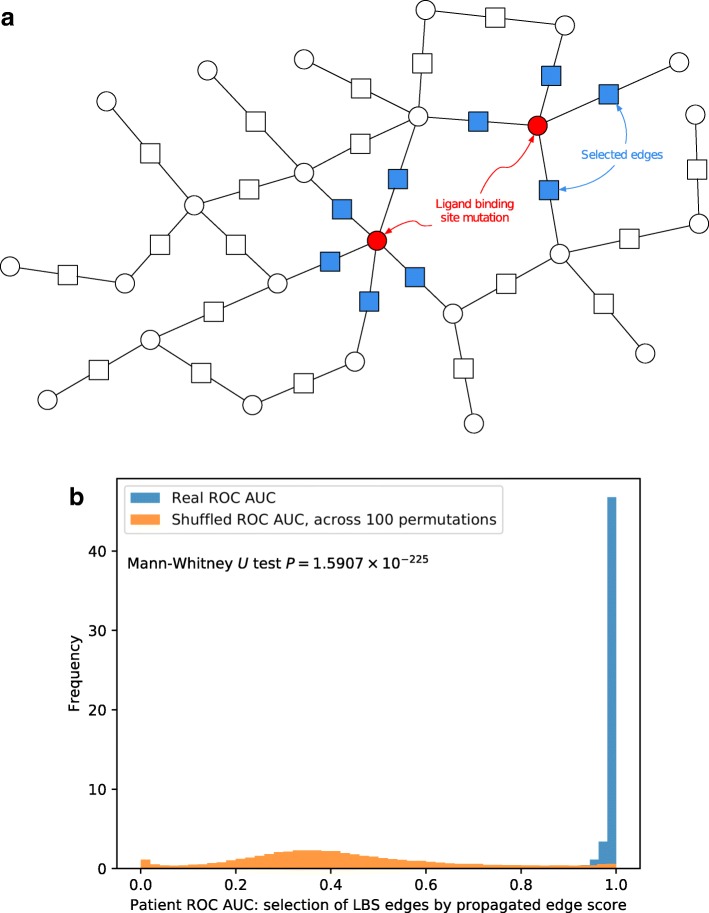
**a** Edge labels for ligand binding site scoring. **b** Histograms of ROC AUC for selection of ligand binding site (LBS) mutation related edges. Scores from real LBS mutations are shown in blue, scores across the 100 shuffled LBS mutation assignments are shown in orange. Frequency values are normalized so that the total area under each histogram sums to 1

Figure 4a shows our assignment of labels to edges: edges are assigned label 1 (shown in blue added node in the middle of the edge) if that edge is adjacent to a ligand binding site mutation (red), and 0 otherwise. This labeling of edges is imperfect; ideally we would label edges as 1 only if that specific interaction is disrupted by a ligand binding site mutation, but the mutLBSgeneDB database [33] does not contain data with this level of granularity.

The total number of patient-model edges in our analysis is 314,727. Of these, only a small fraction are LBS edges, with counts per patient shown in Additional file 1: Figure S3. We consider each of the 389 patients with LBS mutations separately (details of mutation and gene counts in “Methods, and Ligand binding site mutations” sections), rank patients’ edges by propagated mutation scores, and evaluate this ranking through three separate measures: ROC AUC, normalized discounted cumulative gain (nDCG) [39, 40], and Spearman correlation *P*-values. For each of these measures, we compute the real ranking for each patient’s edges, with LBS mutations from the mutLBSgeneDB database, with histograms of ranking measures shown in blue in Fig. 4b and Additional file 1: Figures S4 and S5. We then generate 100 random sets by shuffling LBS assignments and computing the rankings of these random permutations. Note that as with other scale-free networks, shuffling a patient’s LBS mutations can have a large effect on the number of edges labeled 1 (shown in blue in Fig. 4a, since this depends on the degree of the nodes in the network. The performance across all 100 random permutations is shown in orange in Fig. 4b and Additional file 1: Figures S4 and S5. As can be seen, for all evaluation metrics we used the top ranked edges based on network propagated scores are significantly more associated with LBS mutations when compared to a random set of edges. We additionally used the Mann-Whitney *U* test to measure the difference in distributions between our top propagated edges and those obtained via shuffled mutations, for all three measures of the quality of this ranking. The difference between real and shuffled nDCG measures has M–W *P*=3.28×10^−222^, and likewise the ROC AUC and Spearman correlation *P*-value measures produce M–W *P*-values of 7.19×10^−283^ and 6.90×10^−176^, respectively.

Table 1 shows the unique interactions among the top 50 highest-scoring edges across all patients. The rank of each interaction is computed as the highest rank of that edge across all patients. The top-scoring edge here involves *HDAC8*, a class I histone deacetylase which is implicated as a therapeutic target in various diseases, including cancer [41, 42], and tumor suppressors *TP53* [43, 44] and *TP63* [45, 46] both score highly. Cytochrome P450 enzymes such as *CYP2A7* and *CYP2A13* score highly as well, and these genes are implicated in bladder cancer but not normally expressed in breast tissue [47, 48].

**Table 1:** Unique interactions from the top 50 scoring edges based on the smoothed mutational score, pooled across all patients

Gene 1	Gene 2	Prop. score	Top rank	References
*STEAP1B*	*STEAP1*	0.300938	1	[49–51, 57]
*TAS1R2*	*TAS1R3*	0.277285	2	
*SCGB3A2*	*MARCO*	0.244833	3	[52, 53]
*CYP2A7*	*CYP2A13*	0.244117	4	[47, 48]
*CNGB1*	*ABCA4*	0.242088	6	[58, 59]
*PLXND1*	*SEMA3E*	0.229689	12	[60]
*GSTA5*	*GSTA2*	0.211860	14	[61]
*UGT2B15*	*UGT2A3*	0.210076	15	[62, 63]
*CX3CR1*	*CX3CL1*	0.206455	16	[64–66]
*GFRA3*	*ARTN*	0.204744	21	[67–70]
*GRID2*	*GRID2IP*	0.202544	33	[71]
*PLXNC1*	*SEMA7A*	0.199880	34	[60, 72]
*HAS2*	*HAS3*	0.199088	35	[73, 74]
*OBP2B*	*OBP2A*	0.197566	37	[75, 76]
*CD180*	*LY86*	0.195079	39	[77, 78]
*ZNF221*	*ZNF225*	0.193870	42	[79, 80]
*PSPN*	*GFRA4*	0.192038	45	[81, 82]
*LIPE*	*FABP9*	0.190075	47	[83, 84]
*KCNC1*	*KCNC2*	0.189430	48	[85, 86]

References refer to prior information about the involvement of these proteins in breast or other types of cancers. See Additional file 1: Table S2 for complete details and more information

Results for alternative edge weights *w*^′^=*w*/2 are shown in Additional file 1: Figures S7–S9, again with highly significant differences between real and shuffled edge selections (M–W *P*=1.59×10^−225^ for ROC AUC, *P*=5.02×10^−213^ for nDCG, and *P*=4.12×10^−181^ for Spearman correlation *P*-values). We likewise see highly significant differences between real and shuffled edge selections with the STRING network, shown in Additional file 1: Figures S13–S15. These figures show significantly higher ROC AUC and nDCG measures for selection of real LBS edges vs. shuffled LBS assignments (M–W *P*=1.12×10^−230^ and *P*=3.04×10^−228^, respectively), though selection of real LBS edges shows significantly lower Spearman correlation *P*-values than shuffled edge assignments (M–W *P*=1.12×10^−230^).

### Protein structure alteration prediction

The above analysis focused on proteins with known ligand binding mutations. However, as mentioned the LBS database does not identify the interacting partner(s) that may are disrupted by the mutation. To test if we indeed can determine significant pairwise events that affect cancer prognosis we next examined the agreement between our patient specific edge disruption scores, the patient mutation profile and changes in predicted binding affinity between pairs of proteins, using the ClusPro 2.0 [36] tool. ClusPro 2.0 simulates protein docking using sampling of billions of conformations, followed by clustering of the lowest energy structures (Additional file 1: Supporting Methods). We started with 143 interactions which could potentially be simulated based on the availability of structure data for both proteins (“Methods” section). However, only a few of these pairs were actually usable for this analysis. While 98 distinct proteins had at least one structure available in PDB [34], few of these proteins had a comprehensive structure available for the entire protein, without including other molecules in complex. Such structure is required for an accurate docking of a pair. We eventually were able to test 14 pairs.

We used our propagated mutational scores to rank the pairs of proteins for which we could conceivably perform binding predictions, and hypothesized that higher propagated mutation scores would correlate with higher disruption of protein binding. To illustrate this analysis consider that the lowest-scoring (indicating little impact) interaction was the pair (*YWHAG*,*SKP1*), with *YWHAG* harboring a ligand binding site mutation causing amino acid substitution S46C; and the highest-scoring pair, (*PTGIS*,*PTGS2*), with a ligand binding site mutation in *PTGIS* that causes amino acid substitution F293L.

**Fig. 5:**
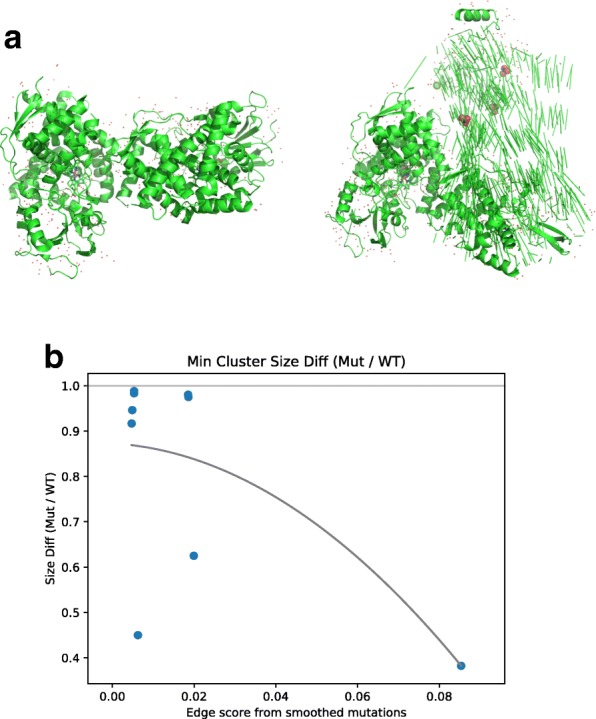
**a** Structure of prostaglandin I2 synthase, product of the *PTGIS* gene. Left: wild type, from PDB structure 2IAG, right: simulation of the impact of the high scoring edge mutation identified for this gene (amino acid substitution F293L). **b** Binding analysis of high and low scoring edges. For each edge we searched for protein structures for the two proteins connected by the edge in PDB. For pairs we found we simulated the impact of the mutation identified for that edge and used the ClusPro 2.0 docking tool to compare WT and mutated binding. Binding scores (*y* axis) represent ratio of maximum protein binding cluster with mutation vs. wild-type proteins. The lower the ratio the bigger the impact of the mutation. Curve is the best fit for a polynomial of degree 2. The curve indicates that as the edge score increases (*x* axis) the impact on binding increases as well

Additional file 1: Figure S6 shows the protein product of the *YWHAG* gene, both wild-type (left) and after using PyMOL [35] to simulate the amino acid change S46C (right). Some small differences in structure are visible, especially in the bottom-left of each structure, but this amino acid substitution shows little effect on the overall structure of the protein. Conversely, Fig. 5a shows the protein produced from the *PTGIS* gene, with left and right showing (respectively) wild-type and the predicted structure after amino acid substitution F293L. As can be seen, in agreement with our assigned higher score, Fig. 5a shows a much more significant alteration of protein structure, consistent with our increased prediction of edge disruption.

We used ClusPro 2.0 to predict binding affinity for all 14 usable pairs of proteins (Fig. 5b). We compute the binding affinity for each of the 14 pairs that we can test, by simulating docking for 1) the two wild-type protein structures, and 2) the simulated effect of the ligand binding site mutation in one protein with the wild-type structure of the other. For each pair of structures (wild-type and wild-type, or wild-type and simulated amino acid substitution), we run ClusPro twice, using each structure for both “receptor” and “ligand” in the ClusPro algorithm. For each {WT⇔WT,mut⇔WT} set of binding possibilities, we compute the ratio of the maximum binding cluster sizes between the mutated pair and the wild-type pair, and consider the minimum of the two ratios for the two assignments of receptor vs. ligand.

Results are shown in Fig. 5b where lower values indicate larger disruption in interaction. We see that the highest-scoring pair, (*PTGIS*,*PTGS2*), has the largest disruption in binding affinity, and that most low-scoring pairs have relatively small disruption in binding affinity. An order-2 polynomial fit for the points is shown in the figure.

## Discussion

In this work, we introduce a method that allows for predicting the disruption of specific interactions in cancer patients using somatic mutation data and condition independent protein interaction networks as input. To do this, we extend traditional network smoothing techniques, which have been previously used to study cancer networks [12, 13, 32], and have also shown promise in the context of network dynamics [15]. Prior network smoothing techniques assigned scores to the nodes in a network based on the measured biological data.(for example mutation status or differential expression). We extended these techniques to assign scores to edges in addition to nodes.

We apply this method to somatic mutation data from the TCGA breast cancer [29] cohort, producing sample-specific scores for each protein-protein edge. We focus on breast cancer in this work due to the large number of samples, but note that our method is general and can be applied to any other cancer types as well. By using somatic mutation data as the prior knowledge vector in network smoothing methods (Supplementary Methods), we quantify the proximity of each protein-protein edge to somatic mutations in individual samples. We show that edges which score highly in at least 20% of samples show significantly higher association with patient survival when compared with random selections of lower-scoring edges. We evaluate the ability of our edge ranking to select interactions involving known ligand binding site mutations [33], and show that we consistently rank LBS mutation incident edges significantly higher than others when compared with random permutations of LBS mutations in each sample. Docking simulations based on the WT and mutants indicate that high scoring edges are indeed more likely to correspond to mutations that can significantly impact protein interactions.

The top 50 pairs ranked by their smoothed mutation scores is presented Table 1 and Additional file 1: Table S1. A number of the pairs and several proteins appear multiple times in different patients. We examined all 38 unique genes in the top 50 interacting pairs for known associations with cancer-related biological processes. As we show in Additional file 1: Table S2, 34 of these 38 genes are indeed known to be associated with at least one type of cancer, most of them with breast cancer and some others with ovarian, prostate or colon cancer. For example, *STEAP1* is overexpressed in many cancers, including breast [49–51]. *SCGB3A2* has been identified as a marker for pulmonary carcinoma in mice and humans [52], and *MARCO* has recently been identified as a possible candidate for targeted antibody therapy in non-small cell lung cancer [53].

## Conclusions

While much of the analysis of coding region mutations focused on their impact on protein structure [17, 54–56], as we show many mutations are actually impacting interactions with key partners. Network smoothing performed across a cohort of patients can provide useful information about such alternation and a mechanistic explanation for the impact of these mutations on cell states. The fact that top scoring edges were significantly correlated with the ability to predict survival is a further indication for the impact that such changes in the interaction networks can cause. With better understanding of underlying causes that lead to cancer, our ability to address some of these issues with appropriate therapeutics would hopefully improve as well.

## Additional file

Additional file 1 Supplementary text. Results of alternative analyses, including using the STRING protein interaction network and using alternative edge weights. (PDF 670 kb)

## Abbreviations

AUC: Area under curve
BRCA: Breast invasive carcinoma
LBS: Ligand binding site
M–W: Mann-Whitney (*U* test)
nDCG: Normalized discounted cumulative gain
PPI: Protein-protein interaction (network)
RCSB: Research Collaboratory for Structural Bioinformatics
ROC: Receiver operator characteristic
TCGA: The cancer genome atlas WT: Wild-type
