# Supplementary material for: Protein interaction disruption in cancer
Source: BMC Cancer. 2019 Apr 23;19:370. doi: 10.1186/s12885-019-5532-5 (PMC6823625; doi:10.1186/s12885-019-5532-5)
Supplement: Supplementary file 1 — Supplementary text. Results of alternative analyses, including using the STRING protein interaction network and using alternative edge weights. (PDF 670 kb) [file 12885_2019_5532_MOESM1_ESM.pdf]

Protein Interaction Disruption in Cancer
(Supplementary Material)

Matthew Ruffalo[1] and Ziv Bar-Joseph[1,2]

[1] *Computational Biology Department, School of Computer Science, Carnegie Mellon University*
[2] *Machine Learning Department, School of Computer Science, Carnegie Mellon University*

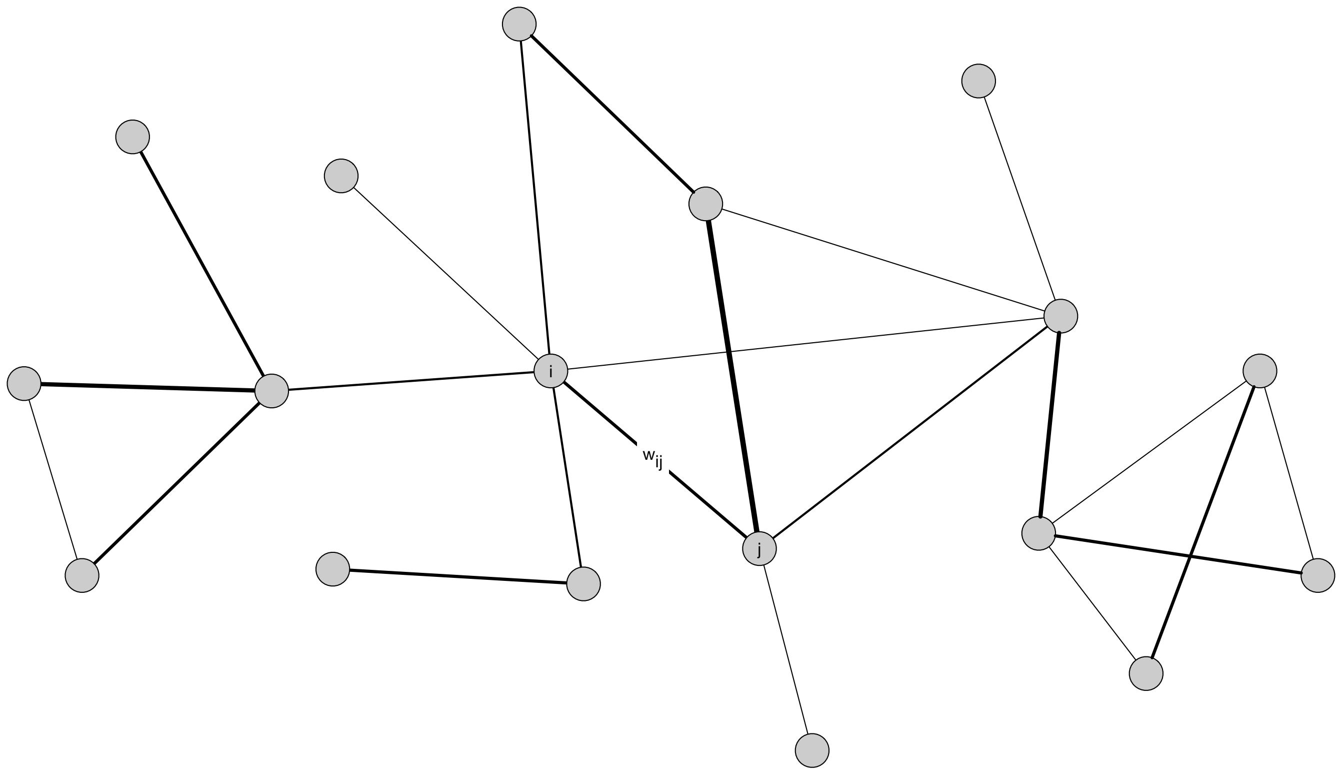

Supplementary Figure S1. Original network

1 Supporting Methods

1.1 Network Adjustment

Supplementary Figure S1 shows an original PPI network with weights w_{ij} on each edge $\{i, j\} \in E$. We adjust this network by adding dummy nodes in the middle of each edge, and connecting these dummy "edge" nodes to the original protein nodes with edges of weight $\sqrt{w_{ij}}$. The adjusted network is shown in Supplementary Figure S2.

We note that our choice of edge weights $w'(u, uv) = w'(uv, v) = \sqrt{w(u, v)}$ preserves the reliability of any path between two nodes s and t representing proteins in the network G, giving the same reliability $r(p_{s't'})$ in G':

$$\begin{aligned} r(p_{st}) &= \prod_{(u,v)\in p_{st}} w(u, v) &(1)\\ &= \prod_{(u,v)\in p_{s't'}} w'(u, uv)w'(uv, v) &(2)\\ &= \prod_{(u,v)\in p_{s't'}} \sqrt{w(u, v)}\sqrt{w(u, v)} &(3)\\ &= r(p_{s't'}) &(4)\end{aligned}$$

1.2 Gene Set Network Smoothing

This network smoothing process uses a normalized edge weight matrix W', computed via Laplacian normalization of the edge weight matrix W: we first construct a diagonal matrix Δ with $\Delta[i, i] = \sum_j W[i, j]$, and compute $W' = \Delta^{-1/2} W \Delta^{-1/2}$. Given a prior knowledge vector Y, we then compute the smoothed vector F using the iterative procedure described by Zhou *et al.* [43]. Starting with $F^{(0)} = Y$, we update F at iteration t as follows:

$$F^{(t)} = \alpha W' F^{(t-1)} + (1 - \alpha)Y \quad (5)$$

This procedure is repeated iteratively until convergence; namely we stop when $\|F^{(t)} - F^{(t-1)}\|_2 < \epsilon$. Note that Laplacian normalization produces a W' with $|\lambda|_{\max} \leq 1$, which is required for this iterative method to converge.

When Y is a binary vector, *i.e.* $Y[u] \in \{0, 1\} \, \forall u \in V$, the value $F[v]$ for a gene v in the smoothed vector F naturally corresponds to a continuous measure of network proximity between v and the "selected" genes $s \in S \subseteq V$ for which $Y[s] = 1$.

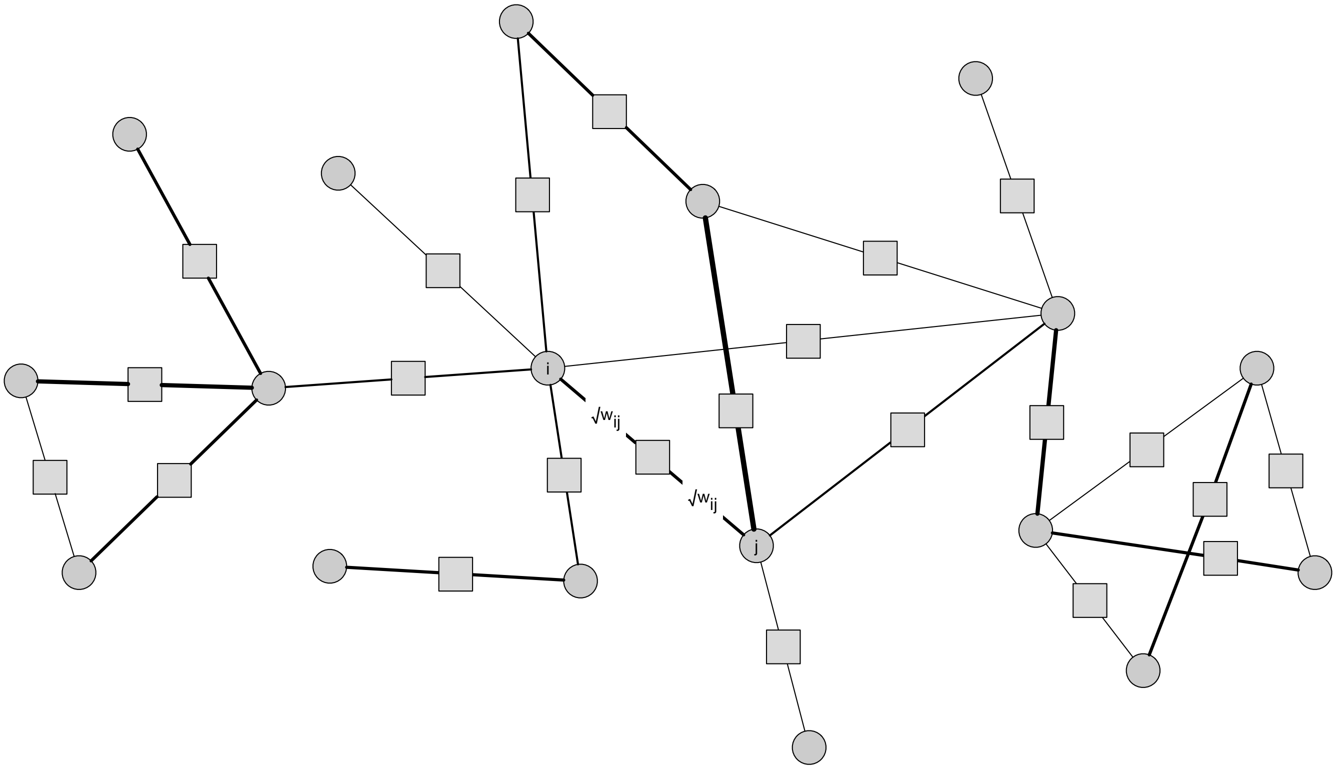

Supplementary Figure S2. Modified network with additional nodes for every edge. Blue circle nodes are proteins in the original network, grey square nodes are dummy nodes that correspond to the edges in the original network.

We therefore this network smoothing method to compute scores of proximity for each gene with respect to the somatic mutations in each sample.

For each aforementioned gene set S, we construct a binary prior knowledge vector Y_S:

$$Y_S[s] = \begin{cases} 1 & \text{if } s \in S \cap V, \\ 0 & \text{otherwise} \end{cases} \tag{6}$$

We then perform network propagation on the vector Y_S, producing a vector F_S. Note that not all genes in the set S are necessarily included in the protein interaction network, and therefore the vectors Y for *e.g.* somatic mutations in a tumor can differ from rows of the somatic mutation matrix M.

We then collect the smoothed vectors into the "propagated" matrix M_P, with $R(M_P) = R(M)$ and $C(M_P) = V$. Intuitively, the propagated matrix M_P contains the per-sample binary vectors of M smoothed over the network. In biological terms, each row of these matrices represents the network proximity of each gene product to mutated genes in that sample. Consequently, the columns of this matrix provides propagated mutation profiles for each gene product across all samples, indicating the proximity of the respective gene product to the products of mutated genes in the respective sample.

1.3 Protein Structure Alteration Prediction

We use the ClusPro 2.0 [18] to simulate protein docking between wild-type and mutated protein structures. ClusPro performs three computational steps to simulate protein docking: (1) rigid body docking by sampling billions of conformations, (2) root-mean-square deviation (RMSD) based clustering of the 1000 lowest energy structures generated to find the largest clusters that will represent the most likely models of the complex, and (3) refinement of selected structures using energy minimization. As recommended by the ClusPro authors, we use the maximum size of an amino acid binding cluster as a measure of binding strength.

2 Results

2.1 Ligand Binding Site Edge Scoring

Supplementary Figure S3 shows the number of ligand binding site (LBS) incident edges per patient, in the TCGA BRCA cohort. Supplementary Figures S4 and S5 show the evaluation of using smoothed mutational scores to rank LBS-incident

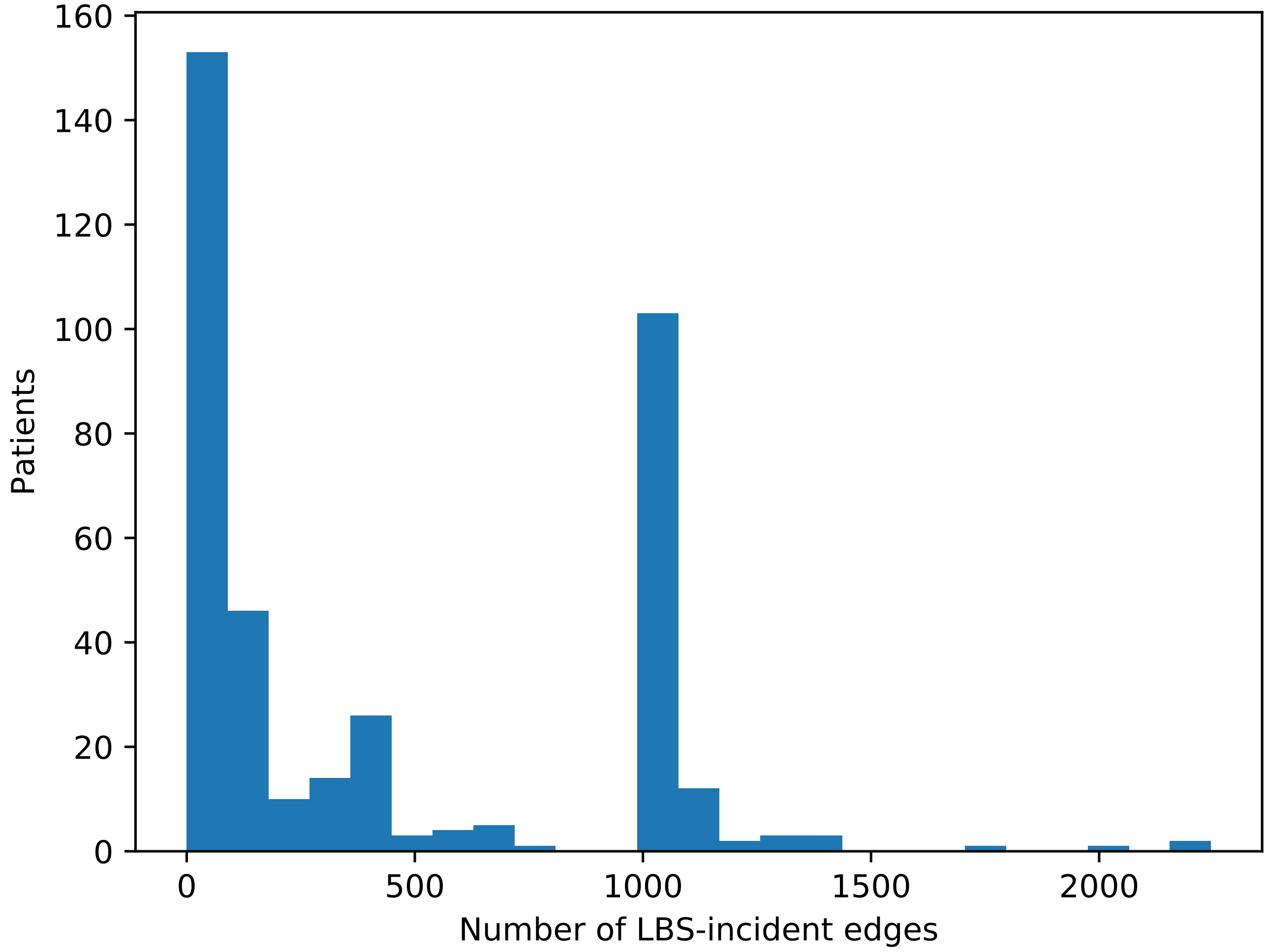

Supplementary Figure S3. Number of LBS edges per patient, computed as shown in Figure 3.

edges; S4 shows the normalized discounted cumulative gain (nDCG) measure, S5 shows Spearman correlation P-values.

2.2 Protein Structure Alteration Prediction

Supplementary Table S2 shows the unique genes present in the top 50 highest-scoring edges, pooled across all patients.

2.3 Alternate Edge Weights

We examine the effect of specific choices of edge scores by repeating our analysis with an alternate method of computing new scores as shown in Supplementary Figure S2. Results ith $w' = w/2$ instead of $w' = \sqrt{w}$ are shown in Supplementary Figures S13 (corresponding to Figure 4b), S14 (corresponding to Supplementary Figure S4), S15 (corresponding to Supplementary Figure S5), and S16 (corresponding to Figure 3).

2.4 STRING Network

In addition to using the HIPPIE v2.0 network, we repeat our analysis using the STRING v10.5 network, using all edges included in the downloadable version of the network. The STRING network includes 4,724,503 edges between 17,179 nodes, with minimum edge weight 0.15.

Results with the STRING network are shown in Supplementary Figures S11 (corresponding to Supplementary Figure S3), S13 (corresponding to Figure 4b), S14 (corresponding to Supplementary Figure S4), S15 (corresponding to Supplementary Figure S5), and S16 (corresponding to Figure 3).

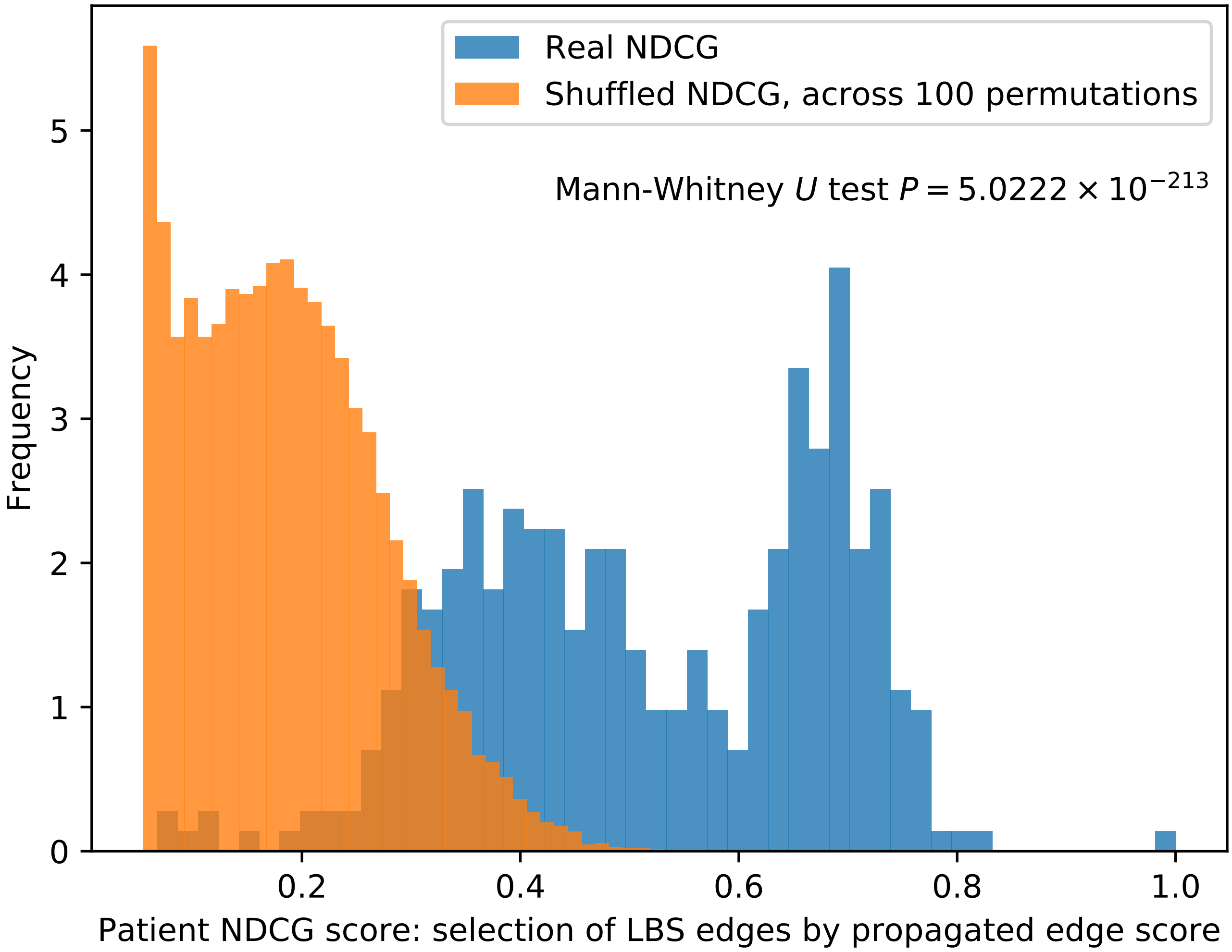

Supplementary Figure S4. Histograms of normalized discounted cumulative gain measures for selection of ligand binding site (LBS) mutation related edges. Scores from real LBS mutations are shown in blue, scores across the 100 shuffled LBS mutation assignments are shown in orange. Frequency values are normalized so that the total area under each histogram sums to 1.

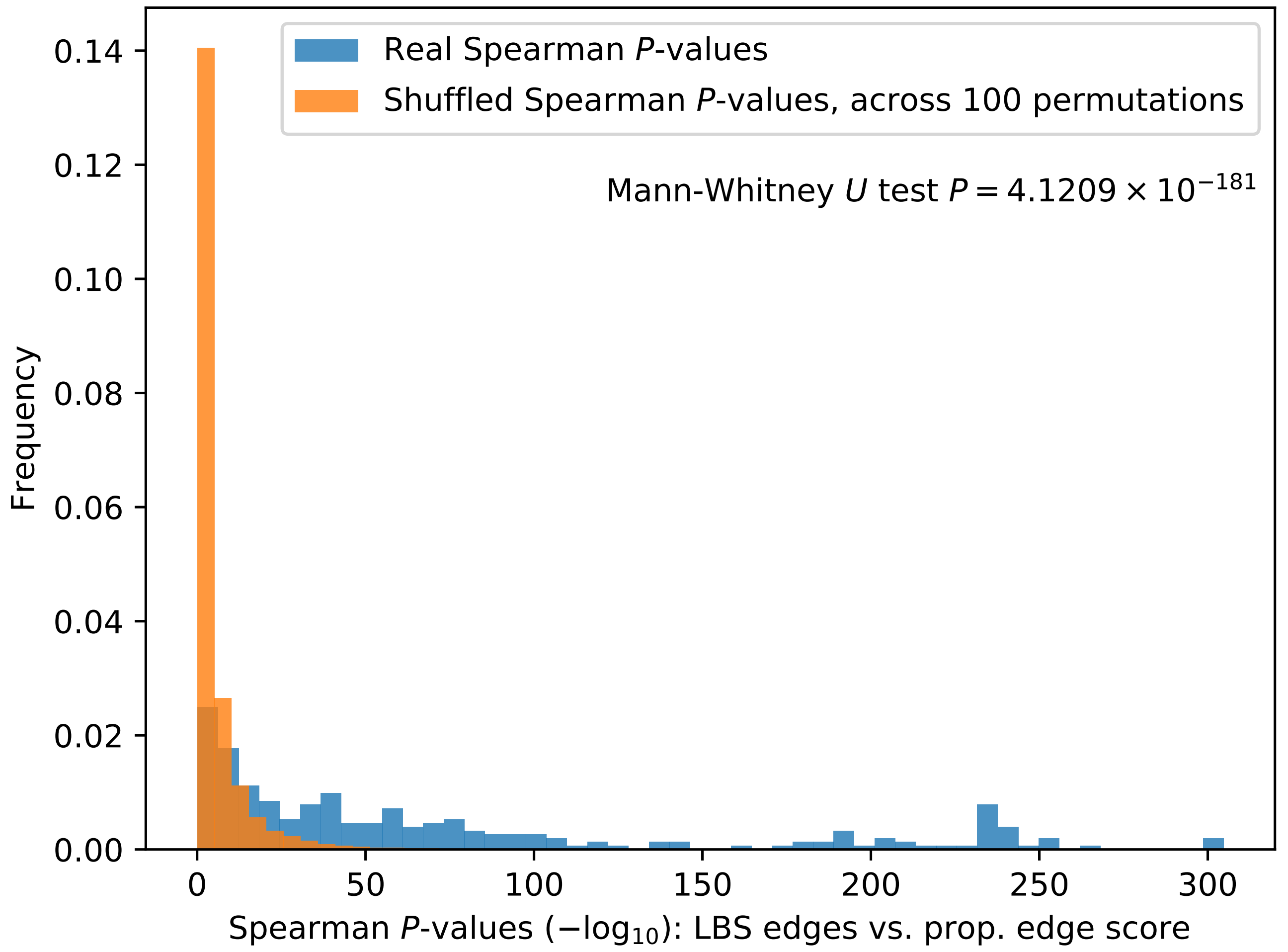

Supplementary Figure S5. Histograms of Spearman correlation P-values for selection of ligand binding site (LBS) mutation related edges. Scores from real LBS mutations are shown in blue, scores across the 100 shuffled LBS mutation assignments are shown in orange. Frequency values are normalized so that the total area under each histogram sums to 1.

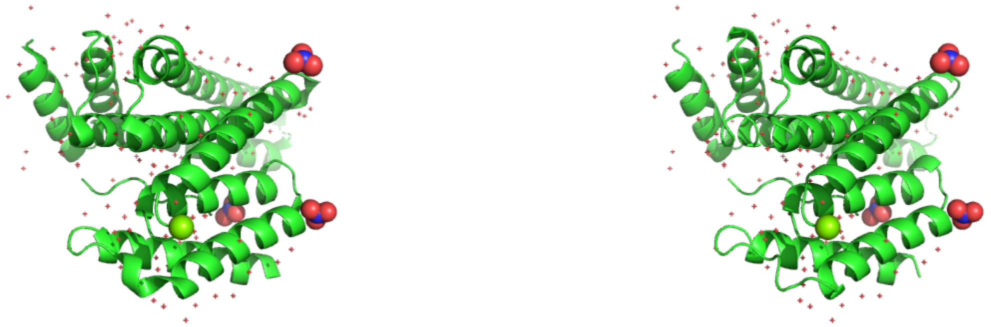

Supplementary Figure S6. Structure of tyrosine 3-monooxygenase/tryptophan 5-monooxygenase activation protein gamma, product of the *YWHAG* gene. Left: wild type, from PDB structure `3UZD`, right: after simulation of amino acid substitution S46C.

**Patient**	**Gene 1**	**Gene 2**	**Prop. Mut. Score**	**Rank**
TCGA-A8-A09I	*ZSCAN2*	*HDAC8*	0.152580	160
TCGA-AN-A046	*ZNF676*	*TP63*	0.140867	451
TCGA-AN-A0XW	*CYP2A7*	*CYP2A13*	0.130999	852
TCGA-AO-A0JD	*CYP2A7*	*CYP2A13*	0.130997	857
TCGA-E2-A14R	*CPA1*	*ARRB1*	0.124980	1767
TCGA-B6-A0RS	*TP53*	*TDRD12*	0.117141	2508
TCGA-BH-A0HA	*ATF7IP*	*PRSS8*	0.113738	3504
TCGA-E2-A10C	*PARP2*	*TPK1*	0.113032	4576
TCGA-A1-A0SG	*PADI2*	*MYC*	0.112586	6442
TCGA-AC-A23H	*PTGIS*	*PTGS1*	0.108385	7314
TCGA-A7-A4SD	*IL11*	*IL11RA*	0.099811	8710
TCGA-E9-A22E	*PRSS2*	*BPI*	0.099311	8779
TCGA-AQ-A04J	*GZMH*	*SSB*	0.098108	9041
TCGA-E2-A154	*PGLYRP3*	*DHTKD1*	0.097734	9224
TCGA-AO-A0JE	*PGLYRP3*	*DHTKD1*	0.097730	9226
TCGA-B6-A0RU	*F2R*	*FABP6*	0.094620	9775
TCGA-AN-A0XW	*SAE1*	*CYP1B1*	0.090848	10888
TCGA-BH-A18M	*ADH7*	*PI4KA*	0.090838	10893
TCGA-E2-A15I	*AKT1*	*DPY19L2*	0.088388	11870
TCGA-E2-A570	*AKT1*	*DPY19L2*	0.088364	11887
TCGA-AR-A251	*MEF2A*	*ZCWPW1*	0.087533	12359
TCGA-AC-A23H	*PTGIS*	*PTGS2*	0.085146	13421
TCGA-BH-A203	*XPO1*	*ZC3H12C*	0.084782	13582
TCGA-BH-A18F	*MROH6*	*XPO1*	0.084685	13618
TCGA-AR-A251	*ZIM2*	*ZCWPW1*	0.083981	13881
TCGA-AN-A0XW	*CYP1B1*	*HSPB2*	0.082346	14988
TCGA-AN-A046	*CFAP57*	*FOS*	0.081983	15297
TCGA-AN-A0XW	*MYD88*	*TLR8*	0.080088	17724
TCGA-AN-A0XW	*TLR8*	*BTK*	0.079912	18257
TCGA-A2-A0EQ	*DDX31*	*TCN1*	0.078767	21412
TCGA-AN-A046	*FOS*	*ATRNL1*	0.077843	22209
TCGA-BH-A18M	*ADH7*	*CAND1*	0.077814	22236
TCGA-AN-A046	*PANK1*	*YWHAZ*	0.075607	23440
TCGA-A2-A0EQ	*TCN1*	*CBL*	0.074200	24454
TCGA-AC-A23H	*PTGIS*	*CAV1*	0.073858	24879
TCGA-BH-A1FM	*ACBD4*	*VAPB*	0.071238	27598
TCGA-AR-A1AU	*IMMT*	*PLA2G15*	0.071222	27616
TCGA-BH-A1FM	*ACBD4*	*VAPA*	0.070687	28194
TCGA-A1-A0SG	*SLC9A4*	*CHP2*	0.069661	29291
TCGA-BH-A0W7	*ADH1A*	*HADH*	0.069390	29534
TCGA-AO-A0J8	*ADH1A*	*HADH*	0.069370	29569
TCGA-BH-A0W7	*ADH1A*	*CSNK2A2*	0.068892	30075
TCGA-AO-A0J8	*ADH1A*	*CSNK2A2*	0.068805	30168
TCGA-C8-A278	*YWHAQ*	*PANK1*	0.068453	30732
TCGA-C8-A278	*PANK1*	*YWHAZ*	0.068424	30779
TCGA-A7-A4SD	*IL11*	*S100P*	0.067424	32186
TCGA-AR-A1AU	*PLA2G15*	*RNF7*	0.067367	32277
TCGA-A1-A0SG	*CHP2*	*SLC9A3*	0.067152	32601
TCGA-AN-A0XO	*ADAMTS5*	*TIMP3*	0.066858	33146
TCGA-A2-A04U	*ADAMTS5*	*TIMP3*	0.066852	33166

Supplementary Table S1. Ranking of the top 50 LBS-adjacent edges.

Gene	Notes	References
ABCA4	Low copy number of *ABCA4* is associated with worse overall survival probability	[10]
ARTN	Has been shown to promote the migration and invasion of pancreatic, endometrial and non-small cell lung cancer cells	[6, 13, 14, 35]
CD180	Part of a seventeen-gene prognostic signature for human HER2+, ER$\alpha-$ breast cancer	[26]
CNGB1	Activation of CNG voltage-gated cation channels has an antiproliferative effect in colon cancer	[16]
CX3CL1	Has been shown to transactivate the EGF pathway and promote breast cancer development	[39]
CX3CR1	Regulates cellular mechanisms involved in proliferation of breast and prostate cancers	[3, 37]
CYP2A13	Cytochrome P450 enzymes are implicated in bladder cancer, but not normally expressed in breast tissue	[12, 32]
CYP2A7		
FABP9	Fatty acid transport and metabolism genes are associated with metastatic progression and poor prognosis of human cancers	[33]
GFRA3	Included in a gene expression signature to distinguish gastric cancer grades and stages	[5]
GFRA4	GDNF family receptor alpha 4 has been shown to be mislocated in glioma cells, correlated with increased glioma proliferation	[22]
GRID2	Possibly associated with prostate cancer outcome in association with H3K27me3 histone marks	[34]
GRID2IP	*No previously known association with cancer*	
GSTA2	*Not* shown to be associated with breast cancer risk	[2, 38]
GSTA5	Tumor cell up-regulation of *GSTA5* shown to be correlated with resistance to chemotherapy agents	[17]
HAS2	Overexpression is implicated in the invasiveness of breast cancer	[41]
HAS3	Overexpression promotes growth of prostate cancer cells	[27]
KCNC1	Part of a marker panel used to identify subtypes of colorectal cancer	[11]
KCNC2	Upregulated in some types of advanced prostate cancer	[25]
LIPE	Increased expression of hormone sensitive lipase has been identified in adipose tissue of cancer patients	[40]
LY86	Downregulated in acute myeloid leukemia, associated with immune response pathways	[30]
MARCO	Possible candidate for targeted antibody therapy in non-small cell lung cancer	[21]
OBP2A	Part of a three-gene signature for prognosis prediction in esophageal squamous cell carcinoma	[9]
OBP2B	Implicated in prostate cancer when fused with *OBP2A*	[1]
PLXNC1	Expression correlated with endothelin receptor B in melanoma	[19]
PLXND1	Sema3E/Plexin D1 signaling in human breast cancers correlates with distant metastatic disease	[28]
PSPN	Implicated in oral cancer progression via RET receptor kinase signaling pathway	[4]
SCGB3A2	Identified as a marker for pulmonary carcinoma in mice and humans	[20]
SEMA3E	Sema3E/Plexin D1 signaling in human breast cancers correlates with distant metastatic disease	[28]
SEMA7A	Up-regulated in oral squamous cell carcinoma	[36]
STEAP1	Overexpressed in many cancers, including breast	[7, 29, 31]
STEAP1B	Related to *STEAP1* in function and structure, and differentially expressed in prostate cancer cell lines	[8]
TAS1R2	*No previously known association with cancer*	
TAS1R3	*No previously known association with cancer*	
UGT2A3	SNP rs17147016 in *UGT2A3* associated with elevated ovarian cancer risk	[24]
UGT2B15	Variant in *UGT2B15* affects tamoxifen response in postmenopausal breast cancer patients	[42]
ZNF221	Differentially expressed in ovarian cancer cells	[15]
ZNF225	Downregulated in ovarian cancer following early chemotherapy	[23]

Supplementary Table S2. Distinct genes shown in Table 2, in the top 50 edges ranked by smoothed somatic mutations across all samples.

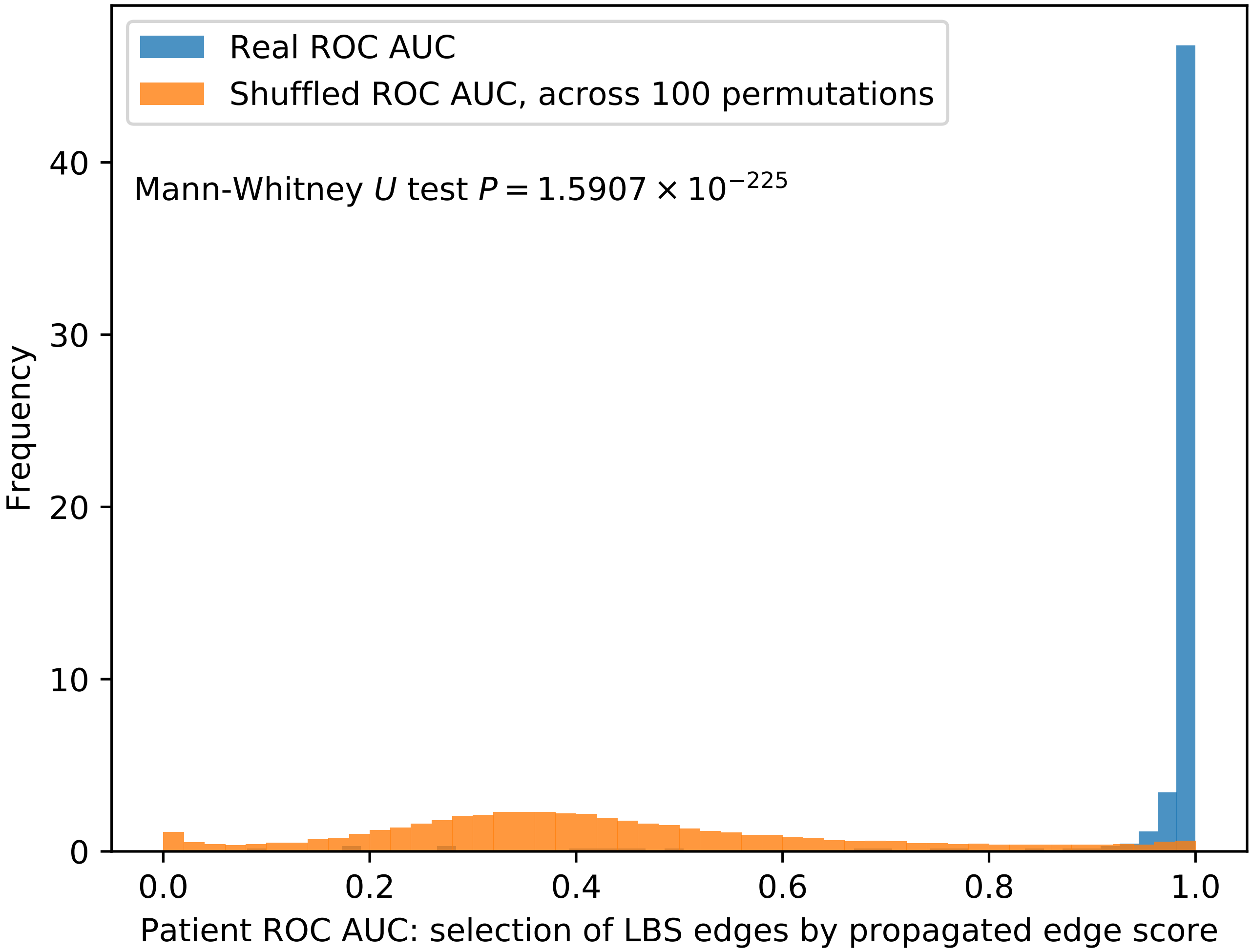

Supplementary Figure S7. Histograms of ROC AUC for selection of ligand binding site (LBS) mutation related edges, similar to Figure 4b but using alternate edge weights.

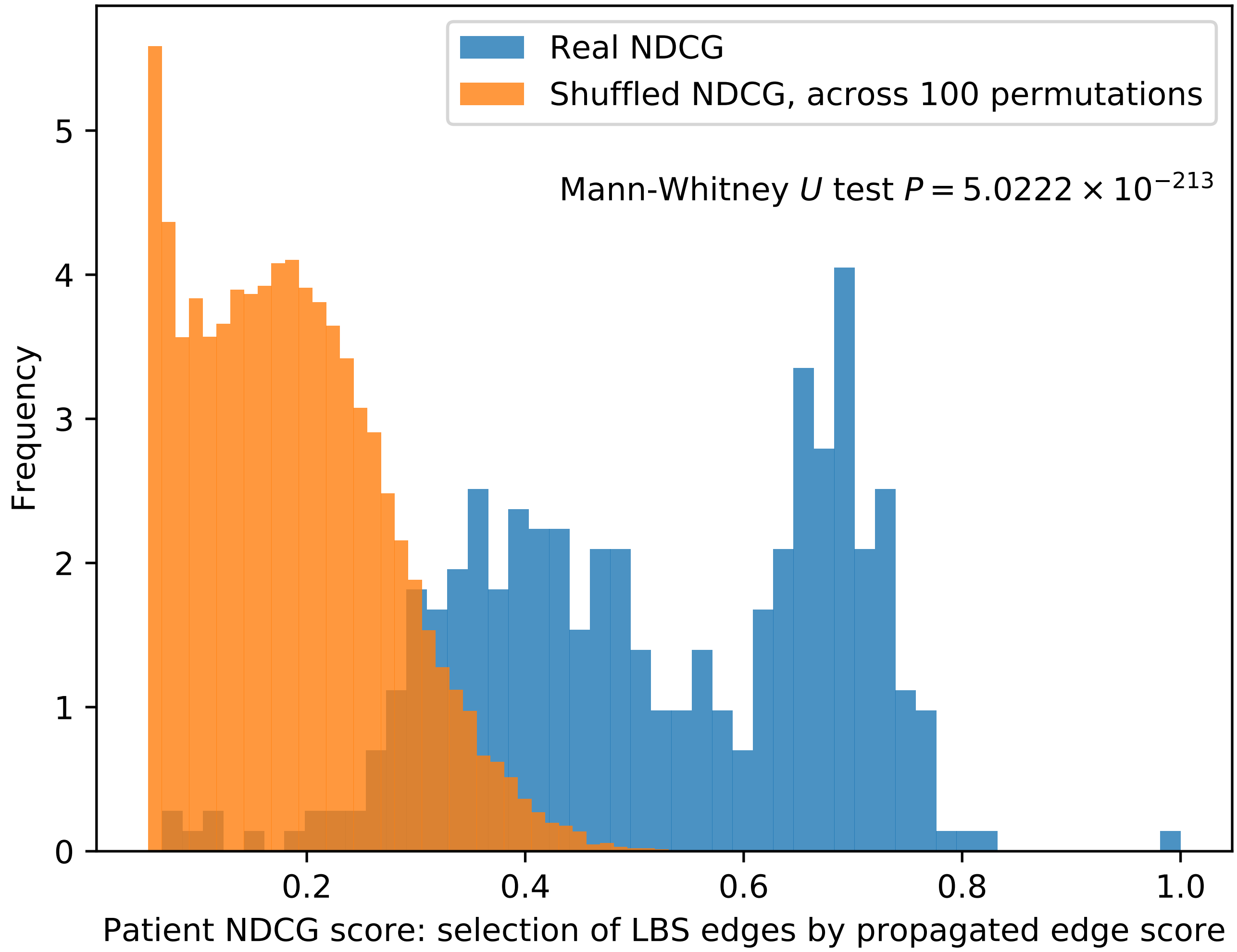

Supplementary Figure S8. Histograms of normalized discounted cumulative gain measures for selection of ligand binding site (LBS) mutation related edges, similar to Supplementary Figure S4 but using alternate edge weights.

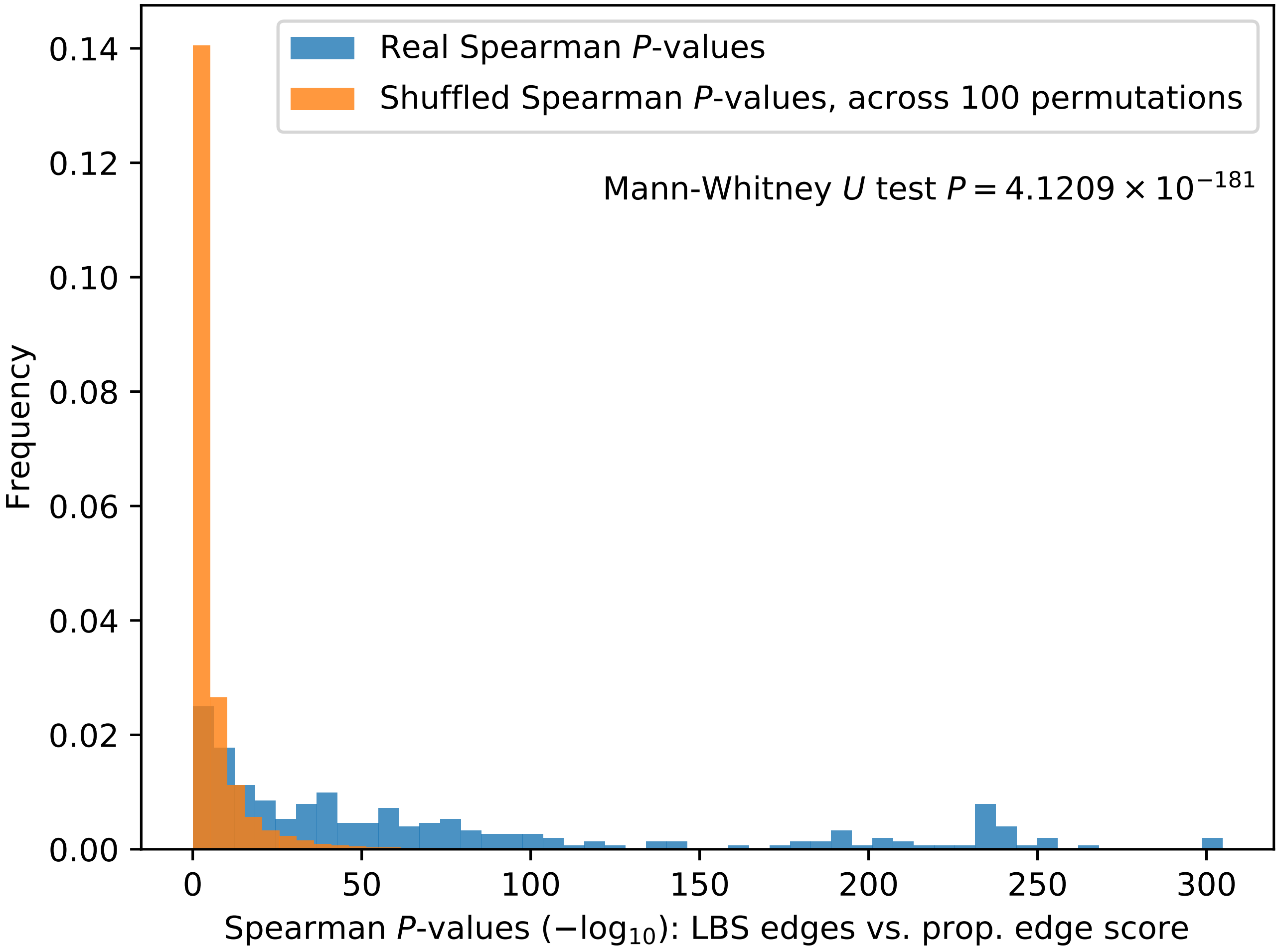

Supplementary Figure S9. Histograms of Spearman correlation P-values for selection of ligand binding site (LBS) mutation related edges, similar to Supplementary Figure S5 but using alternate edge weights.

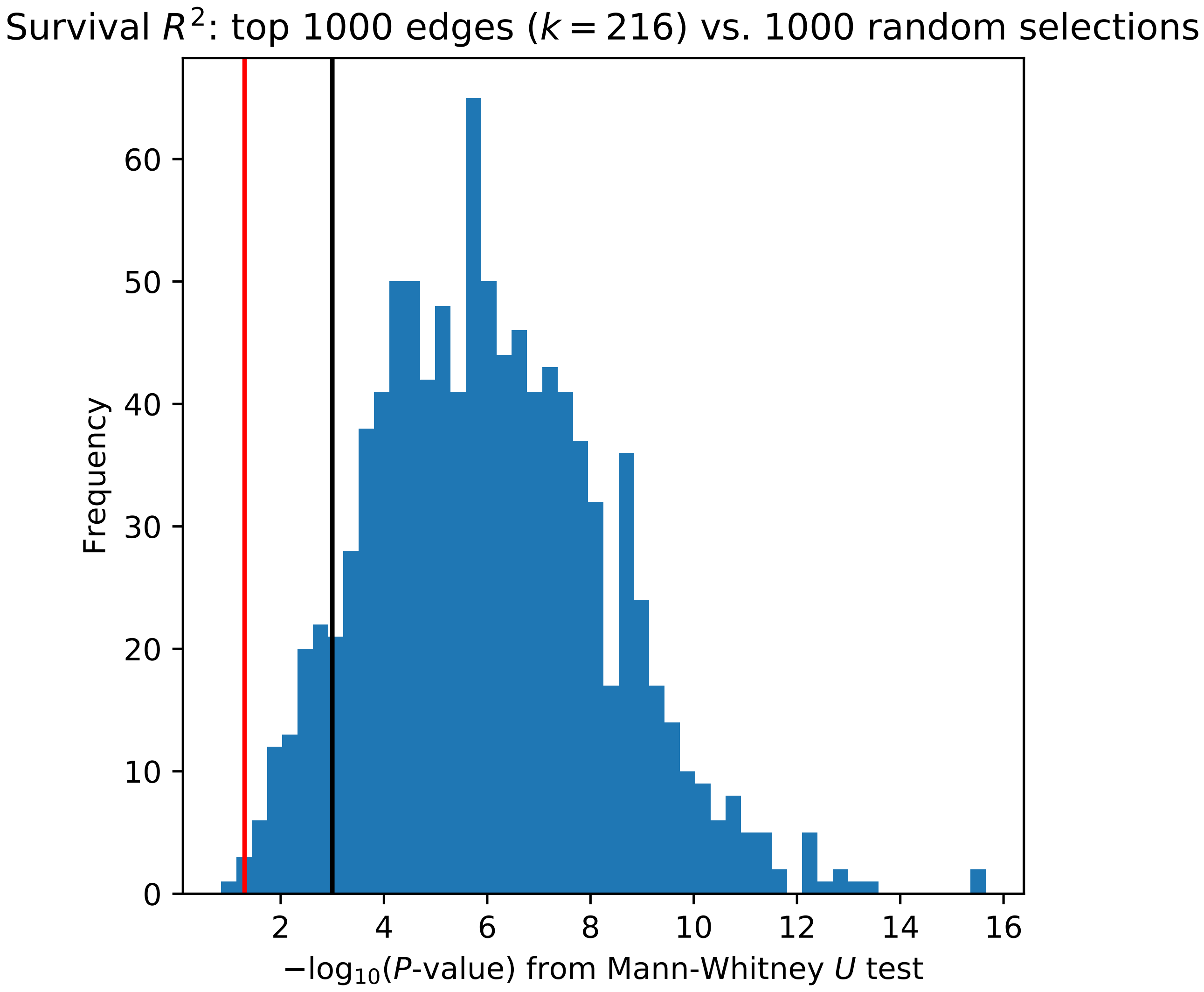

Supplementary Figure S10. Histogram of Mann-Whitney U test P-values, comparing survival R^2 values for top-scoring edges and 1,000 sets of randomly-selected edges, similar to Figure 3 but using alternate edge weights. The red vertical line shows $P = 0.05$, the black vertical line shows $P = 0.001$.

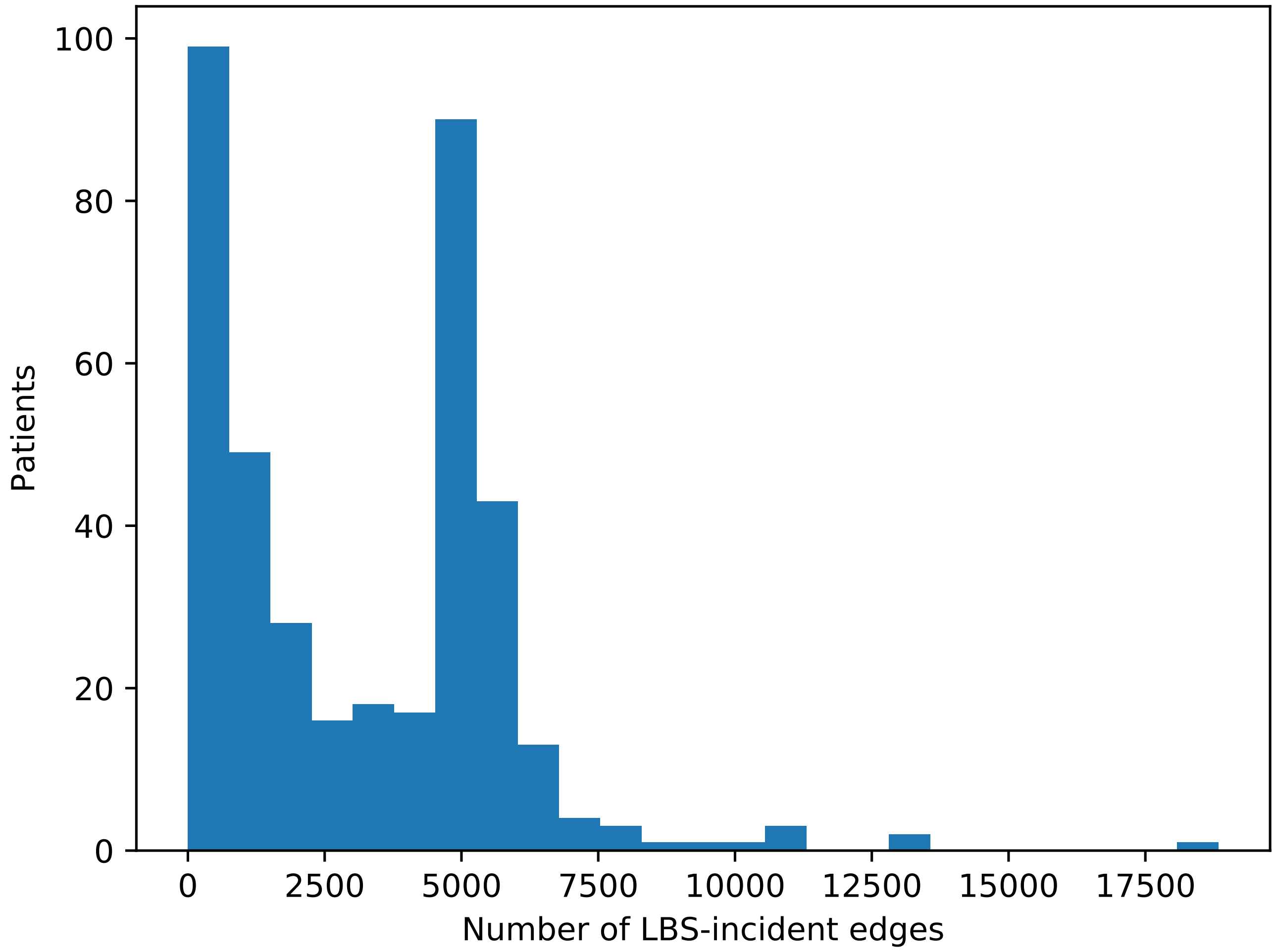

Supplementary Figure S11. Number of LBS edges per patient, similar to Supplementary Figure S3 but using the STRING network.

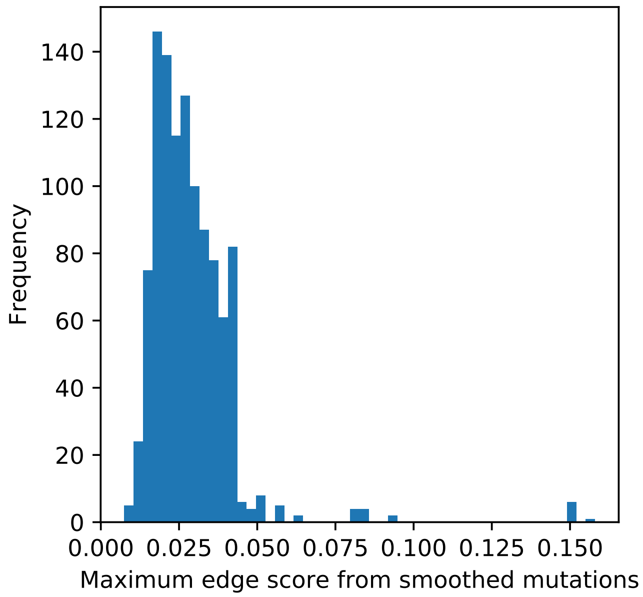

(a) Histogram of maximum edge scores across patients, using the STRING network.

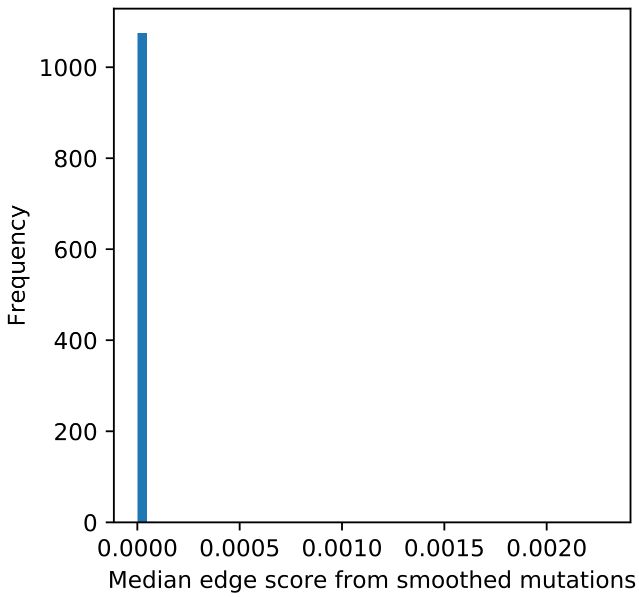

(b) Histogram of median edge scores across patients, using the STRING network.

Supplementary Figure S12. Histograms of propagated edge scores, similar to Figure 3 but using the STRING network. For each patient, scores are collapsed across all edges by computing the median or maximum edge score in that patient. **(a)** shows the distribution of the median edge score in each patient, and **(b)** shows the distribution of the maximum edge score in each patient.

References

[1] I Teles Alves, Thomas Hartjes, Elizabeth McClellan, Saskia Hiltemann, René Böttcher, Natasja Dits, MR Temanni, Bart Janssen, W Van Workum, Peter van der Spek, et al. Next-generation sequencing reveals novel rare fusion events with functional implication in prostate cancer. *Oncogene*, 34(5):568, 2015.

[2] Irena E Andonova, Christina Justenhoven, Stefan Winter, Ute Hamann, Christian Baisch, Sylvia Rabstein, Anne Spickenheuer, Volker Harth, Beate Pesch, Thomas Brüning, et al. No evidence for glutathione s-transferases gsta2, gstm2, gsto1, gsto2, and gstz1 in breast cancer risk. *Breast cancer research and treatment*, 121(2):497–502, 2010.

[3] F Andre, NHJC Cabioglu, H Assi, JC Sabourin, S Delaloge, A Sahin, K Broglio, JP Spano, C Combadiere, C Bucana, et al. Expression of chemokine receptors predicts the site of metastatic relapse in patients with axillary node positive primary breast cancer. *Annals of Oncology*, 17(6):945–951, 2006.

[4] Takao Baba, Yosuke Sakamoto, Atsushi Kasamatsu, Yasuyuki Minakawa, Satoshi Yokota, Morihiro Higo, Hidetaka Yokoe, Katsunori Ogawara, Masashi Shiiba, Hideki Tanzawa, et al. Persephin: A potential key component in human oral cancer progression through the ret receptor tyrosine kinase-mitogen-activated protein kinase signaling pathway. *Molecular carcinogenesis*, 54(8):608–617, 2015.

[5] Juan Cui, Fan Li, Guoqing Wang, Xuedong Fang, J David Puett, and Ying Xu. Gene-expression signatures can distinguish gastric cancer grades and stages. *PloS one*, 6(3):e17819, 2011.

[6] Chaobing Gao, Xingwang Cheng, Xiaohong Li, Busheng Tong, Kaile Wu, and Yehai Liu. Prognostic significance of artemin and gfrα1 expression in laryngeal squamous cell carcinoma. *Exp Ther Med*, 8(3):818–822, Sep 2014. etm-08-03-0818[PII].

[7] Inês M Gomes, Patrícia Arinto, Carlos Lopes, Cecília R Santos, and Cláudio J Maia. Steap1 is overexpressed in prostate cancer and prostatic intraepithelial neoplasia lesions, and it is positively associated with gleason score. In *Urologic Oncology: Seminars and Original Investigations*, volume 32, pages 53–e23. Elsevier, 2014.

[8] Inês M Gomes, Cecília R Santos, and Cláudio J Maia. Expression of steap1 and steap1b in prostate cell lines, and the putative regulation of steap1 by post-transcriptional and post-translational mechanisms. *Genes & cancer*, 5(3-4):142, 2014.

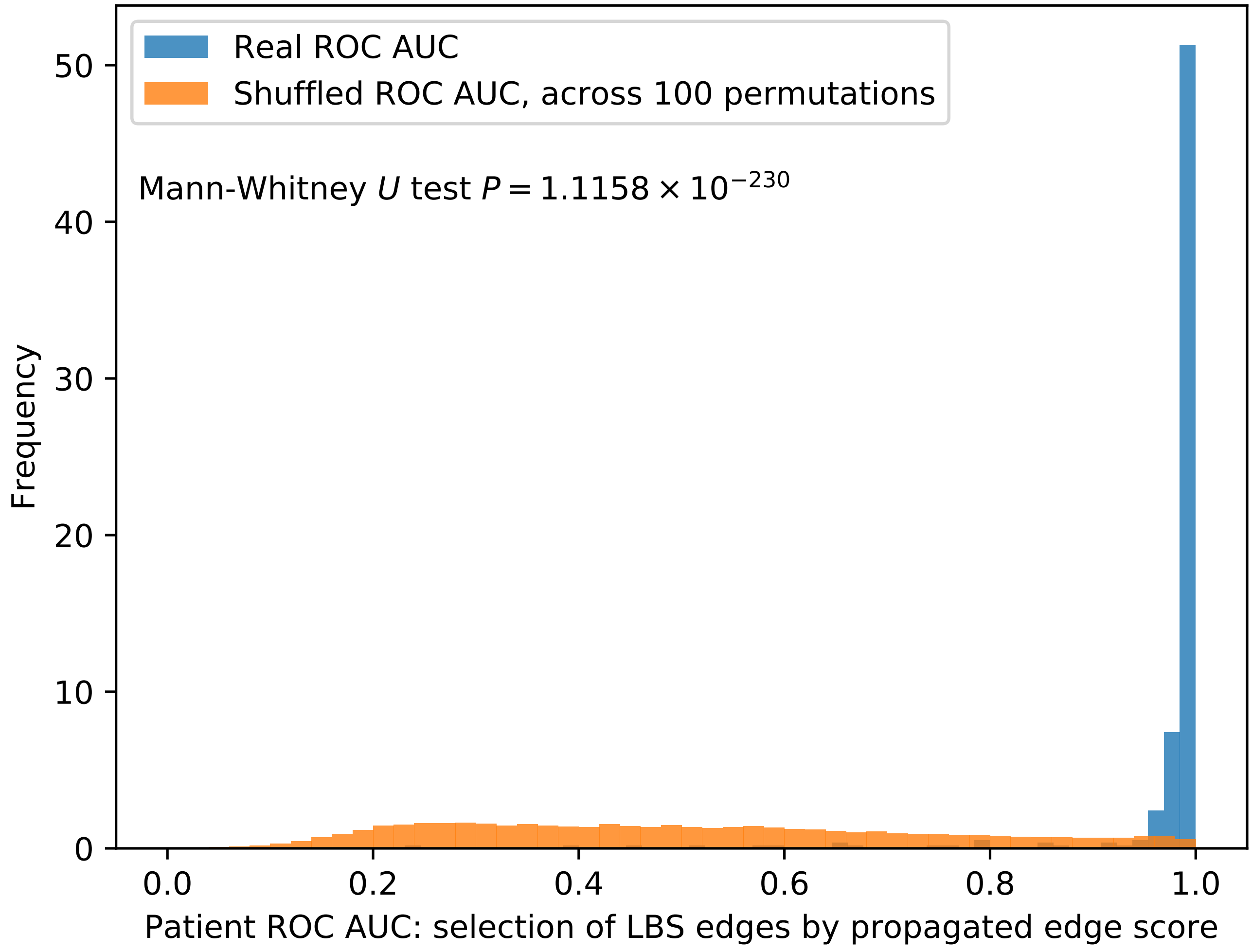

Supplementary Figure S13. Histograms of ROC AUC for selection of ligand binding site (LBS) mutation related edges, similar to Figure 4b but using the STRING network.

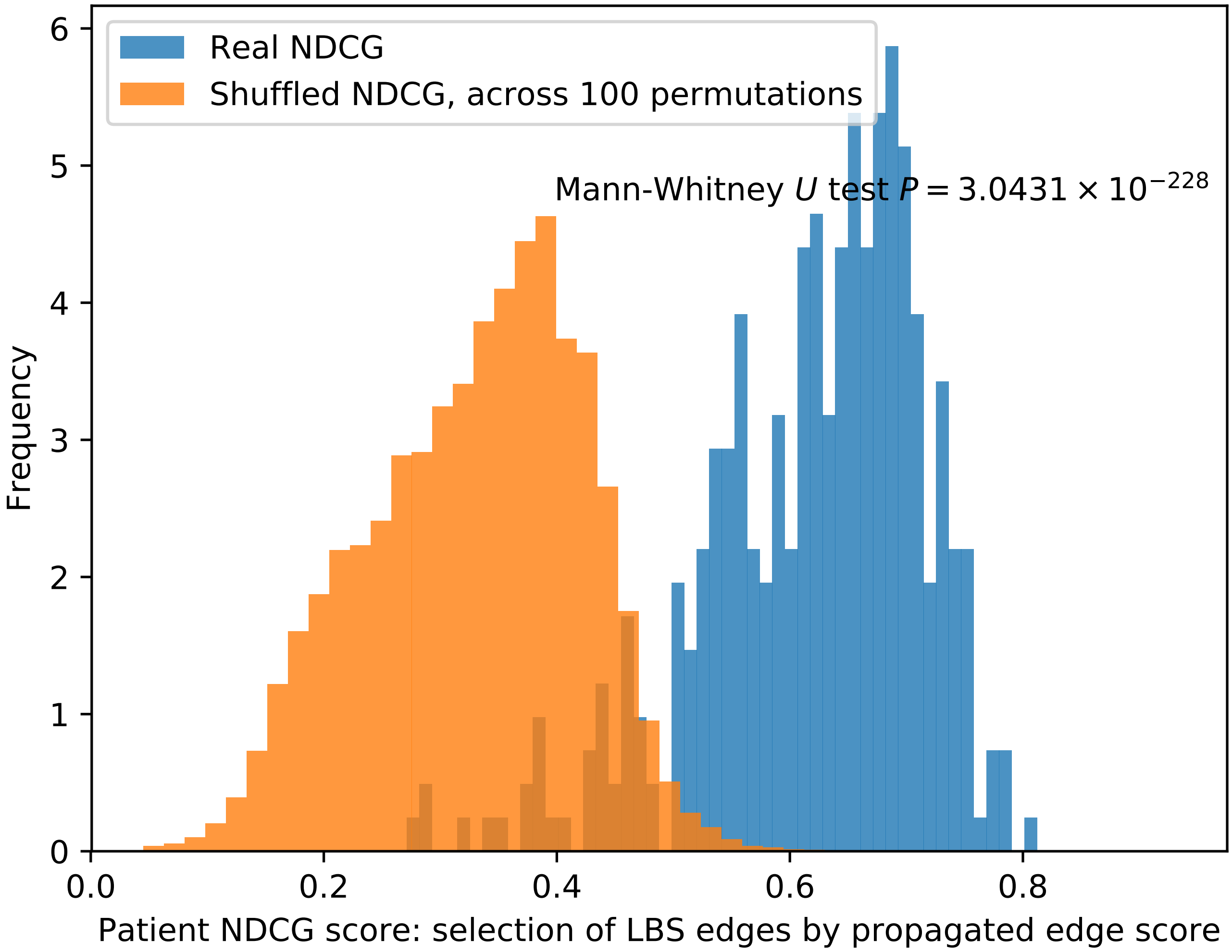

Supplementary Figure S14. Histograms of normalized discounted cumulative gain measures for selection of ligand binding site (LBS) mutation related edges, similar to Supplementary Figure S4 but using the STRING network.

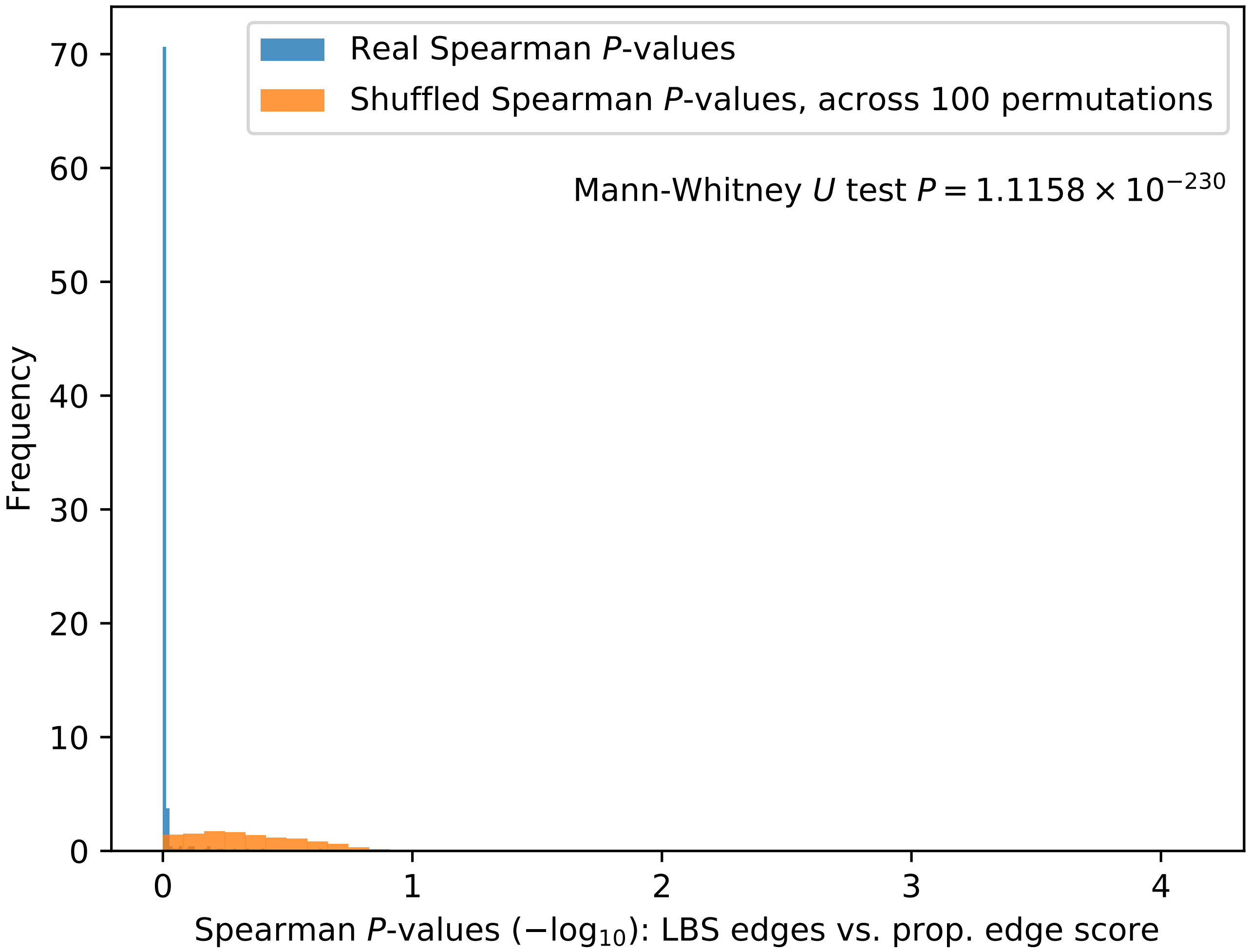

Supplementary Figure S15. Histograms of Spearman correlation P-values for selection of ligand binding site (LBS) mutation related edges, similar to Supplementary Figure S5 but using the STRING network.

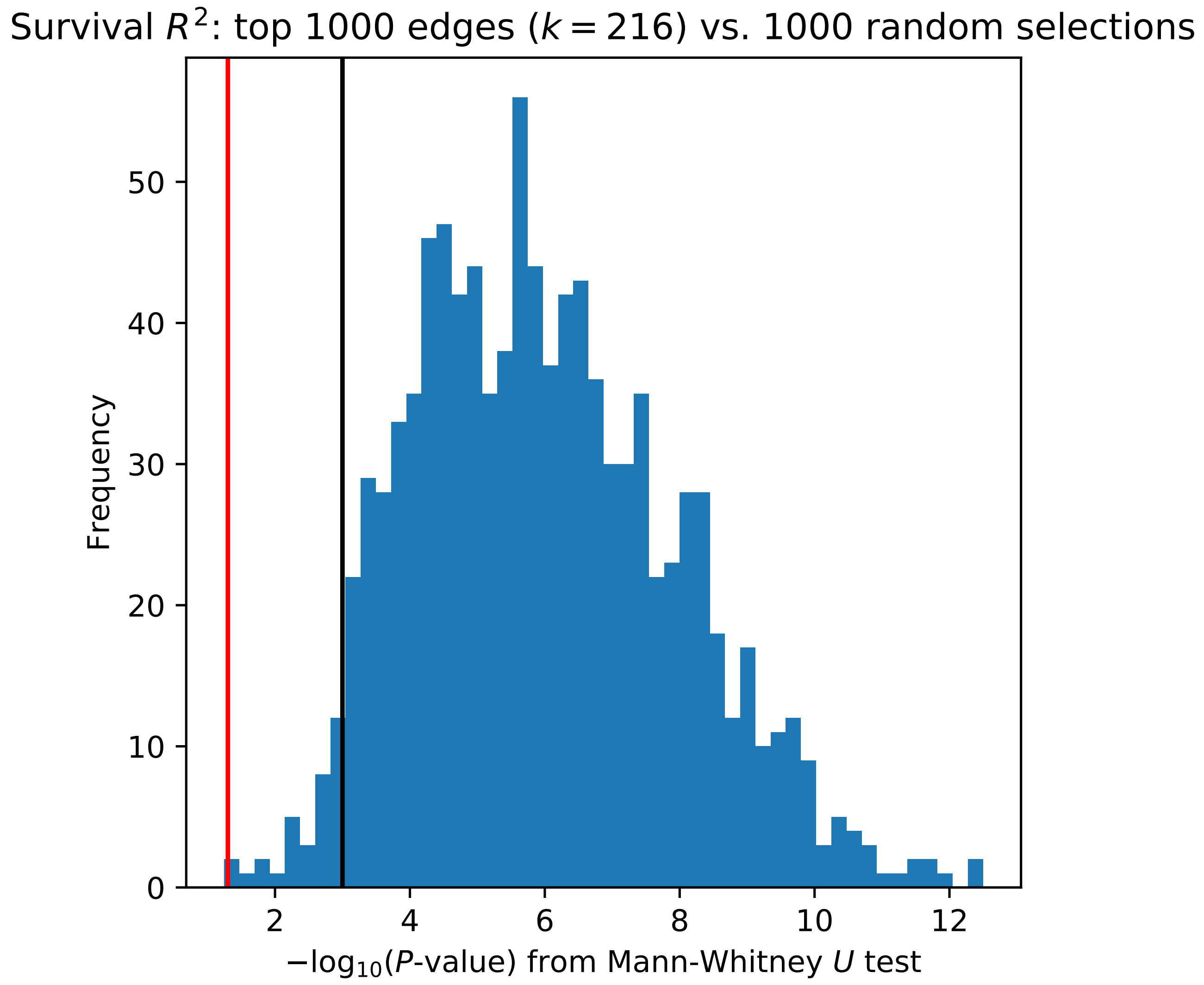

Supplementary Figure S16. Histogram of Mann-Whitney U test P-values, comparing survival R^2 values for top-scoring edges and 1,000 sets of randomly-selected edges, similar to Figure 3 but using the STRING network. The red vertical line shows $P = 0.05$, the black vertical line shows $P = 0.001$.

[9] Jin-Cheng Guo, Chun-Quan Li, Qiu-Yu Wang, Jian-Mei Zhao, Ji-Yu Ding, En-Min Li, and Li-Yan Xu. Protein-coding genes combined with long non-coding rnas predict prognosis in esophageal squamous cell carcinoma patients as a novel clinical multi-dimensional signature. *Molecular BioSystems*, 12(11):3467–3477, 2016.

[10] K Havrysh and R Kiyamova. 14new potential biomarkers for breast cancer prognosis. *Annals of Oncology*, 28(suppl7):mdx508.011, 2017.

[11] Toshinori Hinoue, Daniel J Weisenberger, Christopher PE Lange, Hui Shen, Hyang-Min Byun, David Van Den Berg, Simeen Malik, Fei Pan, Houtan Noushmehr, Cornelis M van Dijk, et al. Genome-scale analysis of aberrant dna methylation in colorectal cancer. *Genome research*, 22(2):271–282, 2012.

[12] Mumtaz Iscan, Tuula Klaavuniemi, Tulay Çoban, Nilgun Kapucuoğlu, Olavi Pelkonen, and Hannu Raunio. The expression of cytochrome p450 enzymes in human breast tumours and normal breast tissue. *Breast cancer research and treatment*, 70(1):47–54, 2001.

[13] Y. Ito, Y. Okada, M. Sato, H. Sawai, H. Funahashi, T. Murase, T. Hayakawa, and T. Manabe. Expression of glial cell line-derived neurotrophic factor family members and their receptors in pancreatic cancers. *Surgery*, 138(4):788–794, 2005. Cited By :47.

[14] Yoshinori Ito, Yuji Okada, Mikinori Sato, Hirozumi Sawai, Hitoshi Funahashi, Takayuki Murase, Tetsushi Hayakawa, and Tadao Manabe. Expression of glial cell line–derived neurotrophic factor family members and their receptors in pancreatic cancers. *Surgery*, 138(4):788–794, 2005.

[15] Stephanie Jutras, Magdalena Bachvarova, Mamadou Keita, Jean-Loup Bascands, Anne-Marie Mes-Masson, John M Stewart, and Dimcho Bachvarov. Strong cytotoxic effect of the bradykinin antagonist bkm-570 in ovarian cancer cells–analysis of the molecular mechanisms of its antiproliferative action. *The FEBS journal*, 277(24):5146–5160, 2010.

[16] Shiva Kazerounian, Giovanni M Pitari, Fawad J Shah, Glen S Frick, Muniswamy Madesh, Inez Ruiz-Stewart, Stephanie Schulz, Gyorgy Hajnóczky, and Scott A Waldman. Proliferative signaling by store-operated calcium channels opposes colon cancer cell cytostasis induced by bacterial enterotoxins. *Journal of Pharmacology and Experimental Therapeutics*, 314(3):1013–1022, 2005.

[17] Sun-Jin Kim, Jang-Seong Kim, Eun Sung Park, Ju-Seog Lee, Qingtang Lin, Robert R Langley, Marva Maya, Junqin He, Seung-Wook Kim, Zhang Weihua, et al. Astrocytes upregulate survival genes in tumor cells and induce protection from chemotherapy. *Neoplasia*, 13(3):286–298, 2011.

[18] Dima Kozakov, David R Hall, Bing Xia, Kathryn A Porter, Dzmitry Padhorny, Christine Yueh, Dmitri Beglov, and Sandor Vajda. The cluspro web server for protein–protein docking. *Nature protocols*, 12(2):255, 2017.

[19] Mayuko Y Kumasaka, Ichiro Yajima, Machiko Iida, Hiro Takahashi, Yuji Inoue, Satoshi Fukushima, Hironobu Ihn, Kozue Takeda, Yuji Naito, Toshikazu Yoshikawa, et al. Correlated expression levels of endothelin receptor b and plexin c1 in melanoma. *American journal of cancer research*, 5(3):1117, 2015.

[20] Reiko Kurotani, Nobue Kumaki, Xu Naizhen, Jerrold M Ward, R Ilona Linnoila, and Shioko Kimura. Secretoglobin 3a2/uteroglobin-related protein 1 is a novel marker for pulmonary carcinoma in mice and humans. *Lung Cancer*, 71(1):42–48, 2011.

[21] Linnéa La Fleur, Vanessa F Boura, Andrey Alexeyenko, Anders Berglund, Victor Pontén, Johanna SM Mattsson, Dijana Djureinovic, Johan Persson, Hans Brunnström, Johan Isaksson, et al. Expression of scavenger receptor marco defines a targetable tumor-associated macrophage subset in non-small cell lung cancer. *International journal of cancer*, 2018.

[22] KiYoung Lee, Kyunghee Byun, Wonpyo Hong, Han-Yu Chuang, Chan-Gi Pack, Enkhjargal Bayarsaikhan, Sun Ha Paek, Hyosil Kim, Hye Young Shin, Trey Ideker, et al. Proteome-wide discovery of mislocated proteins in cancer. *Genome research*, 23(8):1283–1294, 2013.

[23] Sylvain L'Espérance, Magdalena Bachvarova, Bernard Tetu, Anne-Marie Mes-Masson, and Dimcho Bachvarov. Global gene expression analysis of early response to chemotherapy treatment in ovarian cancer spheroids. *BMC genomics*, 9(1):99, 2008.

[24] Dong Liang, Larissa Meyer, David W Chang, Jie Lin, Xia Pu, Yuanqing Ye, Jian Gu, Xifeng Wu, and Karen Lu. Genetic variants in microrna biosynthesis pathways and binding sites modify ovarian cancer risk, survival, and treatment response. *Cancer research*, 70(23):9765–9776, 2010.

[25] Pei-Chun Lin, Eugenia G Giannopoulou, Kyung Park, Juan Miguel Mosquera, Andrea Sboner, Ashutosh K Tewari, Levi A Garraway, Himisha Beltran, Mark A Rubin, and Olivier Elemento. Epigenomic alterations in localized and advanced prostate cancer. *Neoplasia*, 15(4):IN2–IN5, 2013.

[26] Jeff C. Liu, Veronique Voisin, Gary D. Bader, Tao Deng, Lajos Pusztai, William Fraser Symmans, Francisco J. Esteva, Sean E. Egan, and Eldad Zacksenhaus. Seventeen-gene signature from enriched her2/neu mammary tumor-initiating cells predicts clinical outcome for human her2+:er- breast cancer. *Proceedings of the National Academy of Sciences*, 109(15):5832–5837, 2012.

[27] Ningfei Liu, Feng Gao, Zeqiu Han, Xueming Xu, Charles B Underhill, and Lurong Zhang. Hyaluronan synthase 3 overexpression promotes the growth of tsu prostate cancer cells. *Cancer research*, 61(13):5207–5214, 2001.

[28] Jonathan Luchino, Mélanie Hocine, Marie-Claude Amoureux, Benjamin Gibert, Agnès Bernet, Amélie Royet, Isabelle Treilleux, Patrick Lécine, Jean-Paul Borg, Patrick Mehlen, et al. Semaphorin 3e suppresses tumor cell death triggered by the plexin d1 dependence receptor in metastatic breast cancers. *Cancer Cell*, 24(5):673–685, 2013.

[29] Cláudio JB Maia, Sílvia Socorro, Fernando Schmitt, and Cecília RA Santos. Steap1 is over-expressed in breast cancer and down-regulated by 17β-estradiol in mcf-7 cells and in the rat mammary gland. *Endocrine*, 34(1-3):108–116, 2008.

[30] Guido Marcucci, Kati Maharry, Yue-Zhong Wu, Michael D Radmacher, Krzysztof Mrózek, Dean Margeson, Kelsi B Holland, Susan P Whitman, Heiko Becker, Sebastian Schwind, et al. Idh1 and idh2 gene mutations identify novel molecular subsets within de novo cytogenetically normal acute myeloid leukemia: a cancer and leukemia group b study. *Journal of clinical oncology*, 28(14):2348, 2010.

[31] Jerome Moreaux, Alboukadel Kassambara, Dirk Hose, and Bernard Klein. Steap1 is overexpressed in cancers: a promising therapeutic target. *Biochemical and biophysical research communications*, 429(3):148–155, 2012.

[32] Miki Nakajima, Masahiro Itoh, Haruko Sakai, Tatsuki Fukami, Miki Katoh, Hiroshi Yamazaki, Fred F Kadlubar, Susumu Imaoka, Yoshihiko Funae, and Tsuyoshi Yokoi. Cyp2a13 expressed in human bladder metabolically activates 4-aminobiphenyl. *International journal of cancer*, 119(11):2520–2526, 2006.

[33] Aritro Nath and Christina Chan. Genetic alterations in fatty acid transport and metabolism genes are associated with metastatic progression and poor prognosis of human cancers. *Scientific reports*, 6:18669, 2016.

[34] Marjolaine Ngollo, Andre Lebert, Marine Daures, Gaelle Judes, Khaldoun Rifai, Lucas Dubois, Jean-Louis Kemeny, Frederique Penault-Llorca, Yves-Jean Bignon, Laurent Guy, et al. Global analysis of h3k27me3 as an epigenetic marker in prostate cancer progression. *BMC cancer*, 17(1):261, 2017.

[35] Vijay Pandey, Peng-Xu Qian, Jian Kang, Jo K Perry, Murray D Mitchell, Zhinan Yin, Zheng-Sheng Wu, Dong-Xu Liu, Tao Zhu, and Peter E Lobie. Artemin stimulates oncogenicity and invasiveness of human endometrial carcinoma cells. *Endocrinology*, 151(3):909–920, 2010.

[36] Tomoaki Saito, Atsushi Kasamatsu, Katsunori Ogawara, Isao Miyamoto, Kengo Saito, Manabu Iyoda, Takane Suzuki, Yosuke Endo-Sakamoto, Masashi Shiiba, Hideki Tanzawa, et al. Semaphorin7a promotion of tumoral growth and metastasis in human oral cancer by regulation of g1 cell cycle and matrix metalloproteases: Possible contribution to tumoral angiogenesis. *PloS one*, 10(9):e0137923, 2015.

[37] Shannon A Shulby, Nathan G Dolloff, Mark E Stearns, Olimpia Meucci, and Alessandro Fatatis. Cx3cr1-fractalkine expression regulates cellular mechanisms involved in adhesion, migration, and survival of human prostate cancer cells. *Cancer research*, 64(14):4693–4698, 2004.

[38] Susana N Silva, Ana Paula Azevedo, Valdemar Teixeira, Julieta Esperança Pina, José Rueff, and Jorge Francisco Gaspar. The role of gsta2 polymorphisms and haplotypes in breast cancer susceptibility: A case-control study in the portuguese population. *Oncology reports*, 22(3):593–598, 2009.

[39] Manuel Tardáguila, Emilia Mira, Miguel A García-Cabezas, Anna M Feijoo, Miguel Quintela-Fandino, Iñigo Azcoitia, Sergio A Lira, and Santos Mañes. Cx3cl1 promotes breast cancer via transactivation of the egf pathway. *Cancer research*, 2013.

[40] Mary P Thompson, Sandra T Cooper, Bryan R Parry, and John A Tuckey. Increased expression of the mrna for hormone-sensitive lipase in adipose tissue of cancer patients. *Biochimica et Biophysica Acta (BBA)-Molecular Basis of Disease*, 1180(3):236–242, 1993.

[41] Lishanthi Udabage, Gary R Brownlee, Susan K Nilsson, and Tracey J Brown. The over-expression of has2, hyal-2 and cd44 is implicated in the invasiveness of breast cancer. *Experimental cell research*, 310(1):205–217, 2005.

[42] Pia Wegman, Sauli Elingarami, John Carstensen, Olle Stål, Bo Nordenskjöld, and Sten Wingren. Genetic variants of cyp3a5, cyp2d6, sult1a1, ugt2b15 and tamoxifen response in postmenopausal patients with breast cancer. *Breast Cancer Research*, 9(1):R7, 2007.

[43] Dengyong Zhou, Olivier Bousquet, Thomas Navin Lal, Jason Weston, and Bernhard Schölkopf. Learning with local and global consistency. *Advances in neural information processing systems*, 16(16):321–328, 2004.
